# CHiMP: deep-learning tools trained on protein crystallization micrographs to enable automation of experiments

**DOI:** 10.1107/S2059798324009276

**Published:** 2024-10-01

**Authors:** Oliver N. F. King, Karl E. Levik, James Sandy, Mark Basham

**Affiliations:** a Diamond Light Source Harwell Science and Innovation Campus DidcotOX11 0DE United Kingdom; b Rosalind Franklin Institute, Harwell Science and Innovation Campus, DidcotOX11 0QS, United Kingdom; UNSW Sydney, Australia

**Keywords:** crystallization, image classification, object detection, instance segmentation, deep learning, macromolecular crystallography

## Abstract

A group of three deep-learning tools, referred to collectively as CHiMP (Crystal Hits in My Plate), were created for analysis of micrographs of protein crystallization experiments at the Diamond Light Source (DLS) synchrotron, UK. The first tool, a classification network, assigns images into categories relating to experimental outcomes. The other two tools are networks that perform both object detection and instance segmentation, resulting in masks of individual crystals in the first case and masks of crystallization droplets in addition to crystals in the second case, allowing the positions and sizes of these entities to be recorded. The creation of these tools used transfer learning, where weights from a pre-trained deep-learning network were used as a starting point and repurposed by further training on a relatively small set of data. Two of the tools are now integrated at the VMXi macromolecular crystallography beamline at DLS, where they have the potential to absolve the need for any user input, both for monitoring crystallization experiments and for triggering *in situ* data collections. The third is being integrated into the XChem fragment-based drug-discovery screening platform, also at DLS, to allow the automatic targeting of acoustic compound dispensing into crystallization droplets.

## Abstract 2

A description is given of new deep-learning tools that analyse experimental micrographs of crystallization experiments to enable the automation of outcome classification, crystal detection and determination of locations to dispense compounds for fragment-based drug discovery at Diamond Light Source.

## 1. Background and introduction

Determination of the three-dimensional structure of proteins by X-ray crystallography is a widely adopted technique that provides invaluable information to allow the function of proteins to be elucidated. It is also capable of providing empirical evidence for the binding of ligands, such as enzyme substrates, cofactors or drug-like molecules, that may modulate enzymatic activity. Despite the recent revolutions in protein structure prediction by artificial intelligence (AI) such as *AlphaFold* (Jumper *et al.*, 2021 ▸) and cryo-electron microscopy (Cheng, 2018 ▸), X-ray crystallography is still the technique capable of providing the highest resolution atomic coordinate information for these macromolecules, thereby enabling analysis of biochemical reactions and binding interactions that are not discernible using other methods.

X-ray crystallography is reliant on the ability to grow crystals, where the constituent molecules arrange into an ordered lattice that is both regular enough and large enough to form a measurable diffraction pattern when interacting with a beam of high-energy (short-wavelength) photons. Since it is generally energetically unfavourable for large, soluble, macromolecules to pack into an ordered lattice in aqueous solutions, protein crystallization is a rare event. Finding the correct combination of protein concentration, temperature and added chemical components to make this process favourable takes much trial and error (Ng *et al.*, 2016 ▸). As a consequence, protein crystallographers perform hundreds, if not thousands, of experiments, thereby increasing their chances of success. This, in addition to the difficulty and high cost of producing a pure protein solution, leads to experiments being set up on a small scale (with a total volume of a few hundred nanolitres), often with the aid of a liquid-dispensing robot.

In a typical vapour-diffusion crystallization experiment, a solution containing the protein is mixed with a ‘cocktail’ of chemical components in order to form a crystallization drop. Collections of these drops are grouped together on crystal microwell plates that can contain 96 (or multiples thereof) cocktail conditions. Initially, the cocktails are selected from commercial collections that have been collated based upon what has been reported in the literature to have been successful for other proteins (Jancarik & Kim, 1991 ▸). Once an initial ‘hit’ condition has been found, further optimization experiments in the chemical space around this set of components can then be used to improve crystal quality and allow scaling-up of the experiments for subsequent studies.

Knowledge of reproducible conditions for protein crystal growth underpins methods such as fragment-based drug discovery (FBDD; Douangamath *et al.*, 2021 ▸) and macromolecular room-temperature X-ray crystallography (RTX; Fischer, 2021 ▸; Moreno-Chicano *et al.*, 2022 ▸). In FBDD, large numbers of consistent crystals are required for soaking experiments in which large libraries of low-molecular-weight compounds are trialled as binders to the protein in question. These compounds are added to the crystallization drops after the crystals have grown and, after a set time period, X-ray diffraction data are collected for each combination of compound and crystal. This allows the discovery of compound class, binding mode and binding location for compound series that can be subsequently developed into drug leads. For RTX experiments, however, large numbers of smaller crystals are grown in the same droplet or vessel and data collected across multiple crystals are often merged together. Growth of these crystals can be achieved using a variety of methods, for example batch crystallization (Beale *et al.*, 2019 ▸). RTX enables the study of proteins under near-physiological conditions, giving insight into protein dynamics and ligand binding in an environment that is free from cryoprotectants and crystal imperfections caused by the cooling process itself (Thorne, 2023 ▸).

Regardless of the end goal of the experiment, protein crystallization trials can be monitored periodically, either manually or via the use of a robotic imaging microscope. In the case of vapour-diffusion experiments, these robotic ‘imagers’ incubate the experimental plates of crystallization drops, identified by a unique barcode, and extract them from storage using a motorized arm before capturing images of the drops using a digital microscope camera. Collection of these images (or micrographs) takes place on a schedule of pre­defined timepoints that may cover a period of days or months. The combination of a large number of experiments (drops) and a number of imaging timepoints leads to the creation of hundreds of microscope images that need inspection either by an expert or by an automated system that is able to achieve the accuracy of an expert.

Once crystals have been grown, the subsequent process of data collection for macromolecular X-ray crystallography (MX) has become increasingly automated, particularly at synchrotron experimental stations. In the case of single-crystal experiments, crystals are first mounted onto standardized pins (normally by humans), and robotic arms are then used to change samples that are stored in cryo-cooled dewars (Cipriani *et al.*, 2006 ▸; Lazo *et al.*, 2022 ▸; McAuley *et al.*, 2015 ▸; Wasserman *et al.*, 2015 ▸); this removes the need to approach the X-ray beam shutter during the allocated experimental time and perform subsequent safety checks before opening the shutter again. This, when coupled with faster, more sensitive detectors, leads to higher sample throughput as well as the ability to collect data remotely. To aid this automation, a number of semi-automated as well as fully automated solutions have been created to centre the crystal in the X-ray beam; these include X-ray-based centring approaches (Song *et al.*, 2007 ▸) and image-based approaches (Pohl *et al.*, 2004 ▸), some of which use deep learning (Ito *et al.*, 2019 ▸; Schurmann *et al.*, 2019 ▸). For RTX a different approach can be taken, with data collection taking place *in situ* within the environment where the crystals are grown (Healey *et al.*, 2021 ▸; Ren *et al.*, 2020 ▸; Sanchez-Weatherby *et al.*, 2019 ▸); this removes the need to manipulate the samples, which in turn reduces the need for human intervention to collect data.

At DLS, the automated VMXi beamline facility (Sanchez-Weatherby *et al.*, 2019 ▸) allows RTX experiments to be run in a fashion that minimizes the need for any intervention by the scientific users of the beamline in the data-collection process. Crystallization experiments are set up by a liquid-handling robot and the resulting crystallization microplates are stored in an imaging incubator that is located at the beamline itself. The plates are then imaged periodically on a predefined schedule. Firstly, crystal location coordinates for data collection are determined from these images and recorded in the ISPyB laboratory information-management system (LIMS; Delagenière *et al.*, 2011 ▸). Then, after the user has selected from the list of points and chosen a recipe for data collection, the crystallization plate is automatically transferred to the beamline imaging hutch. Data collection is performed *in situ*, with the microplate mounted directly on a goniometer. Any resulting diffraction patterns are then processed by way of automated software pipelines that include the capability to merge data sets from multiple crystals (Gildea *et al.*, 2022 ▸). Prior to the work described in this study, both the classification of experimental outcomes and the identification of coordinates for data collection required the scientific user to log into the SynchWeb interface to ISPyB (Fisher *et al.*, 2015 ▸) and browse through all of the microscope images before marking the location of points for data collection manually; a time-consuming task.

At the XChem facility, which is also located at DLS in addition to the Crystallisation Facility at Harwell, FBDD campaigns are run on proteins of interest using hundreds of low-molecular-weight compounds (fragments; Douangamath *et al.*, 2021 ▸). Rather than co-crystallizing the compound with the protein, many crystals of the same protein are grown in the absence of compounds and the fragments are subsequently soaked into the crystals. Generally, after a compound has been dispensed into one drop that contains one or more crystals, the molecule is allowed time to diffuse into the crystal lattice before the crystals are manually mounted on pins and cryo-cooled for X-ray data collection. The compounds are dispensed in the form of high-concentration solutions in dimethyl sulfoxide (DMSO) and precisely targeted into the crystallization drop using acoustic dispensing. The targeting location is manually determined upon the inspection of a micrograph of the crystal drop by the scientist, who will try to choose a point within the drop that is far enough from crystals of interest to prevent damage, either from a high local concentration of DMSO or from the physical stresses associated with bombardment of the crystal with a drop of foreign liquid.

In this study, alongside providing source code and training data (see the Data availability section), we describe the creation of two deep-learning tools for the DLS VMXi beamline that simplify the task of browsing images and selecting coordinates for data collection. These tools can potentially replace the need for any user intervention at all, thereby removing a major bottleneck in the workflow. The first is an image-classification network given the name the CHiMP (Crystal Hits in My Plate) Classifier, which outperforms the best classifier network from the literature (Bruno *et al.*, 2018 ▸) on our in-house images. The second is an image object-detection and instance-segmentation network named VMXi CHiMP Detector. Additionally, we describe a third software tool, XChem CHiMP Detector, an image object-detection and instance-segmentation network that allows automated calculation of coordinates for acoustic dispensing of compound solutions into crystallization drops. XChem CHiMP Detector facilitates the automated dispensing of high-concentration fragment compounds for FBBD campaigns that can involve thousands of compound and crystal combinations.

### 1.1. Background on the automated classification of experimental images

Over the past 40 years, a wide variety of techniques have been investigated to automate the monitoring of crystallization trials, with the methodology used reflecting changes in hardware and software capabilities during this period. Comparisons of the effectiveness of these analyses are often difficult, with each institution having its own scoring system for experimental outcomes, which then leads to an arbitrary number of categories (for example different types of protein precipitation, different sizes and classes of crystals, and other phenomena such as phase separation or clear drops) that each image is labelled with. In addition, human expert scorers often disagree on the categories assigned to the same set of images, with a reported rate of ∼70% agreement between 16 crystallographers in one study (Watts *et al.*, 2008 ▸) and an agreement rate of between 50% and 70% between three researchers in another (Milne *et al.*, 2023 ▸). This disagreement not only reflects the difficulty of categorizing a continuum of outcomes (for example ‘Light Precipitate’ versus ‘Heavy Precipitate’), but also the convention of only assigning one label to an image which may represent multiple outcomes (for example, an image of a drop containing spherulites and contamination by a clothing fibre could be labelled as either ‘Spherulites’ or ‘Contaminated’).

#### 1.1.1. Classification metrics

A range of metrics are used to report the effectiveness of algorithms that automate the classification of images. Accuracy is widely used, but can be a misleading metric for un­balanced data sets such as those from crystallization experiments. Images that record successful crystallization may be a minority class in the raw data; however, conversely, they are often the majority class in images that have been scored. This is because scientists will tend to label images that contain crystals and ignore those that do not. Here, we will focus on the precision, the recall and the *F*1 measure of correctly classifying images into the ‘Crystals’ class to compare classification performance across the literature as well as to assess the performance of the tools described in this study. This class is a superset of all categories of crystalline outcomes excluding crystalline precipitate.

Precision is defined as
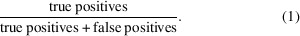


Recall is defined as
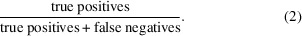


The *F*1 measure is the harmonic mean of precision and recall:
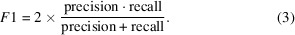


As shown in equation (1), precision is the ratio of correctly predicted positive observations to the total predicted positives, which means that when a methodology with a high precision score predicts a positive result, it is likely to be correct. A high precision can also be obtained when the method is conservative in its positive predictions, missing the true positives in order to avoid false positives. The recall metric (equation 2), however, measures the fraction of correctly predicted positive instances retrieved by the method (the sensitivity). Methods with higher recall values capture a larger portion of the true positives, minimizing false negatives. There is a trade-off between optimizing a method for each of these two measures, with a more precise method missing some actual positive instances, resulting in a lower recall, and a method with a high recall capturing more false positives along with the true positives, reducing precision. The *F*1 score (equation 3) is a harmonic mean of precision and recall and balances both measures in one metric.

#### 1.1.2. Previous investigations of automated classification

The first investigations applied image convolutions to enhance vertical and horizontal edges (Ward *et al.*, 1988 ▸) before further interpretation that used a nearest-neighbour line-tracking algorithm, or a Hough transform (Hough, 1962 ▸), to detect straight lines (Zuk & Ward, 1991 ▸). Feature extraction from images coupled with statistical techniques such as linear discriminant analysis (Cumbaa *et al.*, 2003 ▸; giving a precision of 0.24, a recall of 0.66 and an *F*1 of 0.35 for ‘Crystals’) or self-organizing feature maps (Kohonen, 1982 ▸; Spraggon *et al.*, 2002 ▸) were subsequently explored.

Several studies focused on extracting image-texture information for feature generation (Cumbaa & Jurisica, 2010 ▸; Liu *et al.*, 2008 ▸; Ng *et al.*, 2014 ▸; Watts *et al.*, 2008 ▸). Machine-learning classifiers were trained using these features. For example, Cumbaa & Jurisica (2010 ▸) used 1492 features to train a ten-category random forest ensemble that achieved a classification precision of 0.64, a recall of 0.71 and an *F*1 of 0.67 for the ‘Crystals’ class. Another method involved convolution with a bank of filters to assign ‘texton’ (texture prototype) values to pixels, creating histograms of texton frequencies (Ng *et al.*, 2014 ▸). These histograms were used to train a random forest ensemble and the posterior probability output by the ensemble was used to rank images based on the likelihood of containing ‘interesting’ features, aiding scientists in efficient image selection.

The rise of convolutional neural networks (CNNs), especially deeper models (with more trainable layers), has removed the need for the extraction of human-designed features since the model is able to adapt or ‘learn’ new convolutional kernels which can extract appropriate features. The pioneering CNN for classifying crystallization trial images, CrystalNet, was a seven-layer custom network (Yann & Tang, 2016 ▸). It was trained on a set of 68 155 images (Snell *et al.*, 2008 ▸) in order to classify images into ten classes. The resulting precision, recall and *F*1 metrics on the validation set were 0.81, 0.77 and 0.79, respectively, for the ‘Crystals’ class. The subset of data used for training consisted of images from microbatch-under-oil experiments where three experts independently agreed on the image class. This differs from a real-world data set, where the image class is often ambiguous.

In a subsequent investigation by Ghafurian *et al.* (2018 ▸), CrystalNet was tested on an internal data set of 486 000 images at Merck. They found that the accuracy of the model was 73.7% on these data, as opposed to the 90.8% reported in the original study. The authors then went on to train a number of different CNN architectures on their data set, again using ten image categories. During training, they compensated for the class imbalance: images containing crystals were a minority class, with nine times more images that did not contain crystals (since successful crystallization is a rare event). This compensation was performed using image augmentations to increase the size of their training data, generating images in inverse proportion to the size of each class of image. The final training data set had over a million images. They found that a deep ResNet model (He *et al.*, 2016 ▸) with 56 trainable layers gave the best results, with a recall of 0.94 when classifying the test set of >200 000 images.

The next, and arguably the most high-profile, CNN to be created for crystallization-micrograph classification was that from the Machine Recognition of Crystallization Outcomes (MARCO) initiative (Bruno *et al.*, 2018 ▸; https://marco.ccr.buffalo.edu/). In this work, a customized variant of the 42-layer Inception v3 CNN architecture was trained on a set of 415 990 images that were collected from several organizations, including GSK and Merck. Rather than a ten-class classification system, the MARCO network outputs one of four labels, namely ‘Crystals’, ‘Clear’, ‘Precipitate’ or ‘Other’. For the ‘Crystals’ class, MARCO achieves a precision of 0.94, a recall of 0.91 and an *F*1 measure of 0.93 on the validation set of images. In addition, the authors also created a test set of images of sitting-drop experiments solely from their institution that had been hand-scored by an expert. In this set of images, the network achieved a precision of 0.78, a recall of 0.87 and an *F*1 score of 0.82. MARCO has been seen as the state of the art in recent years and has been incorporated into commercial software for crystal plate imagers (Formulatrix, 2019 ▸).

The MARCO data set is openly available (https://marco.ccr.buffalo.edu/) and has been used to train and evaluate the models in other studies. One approach used two CNNs (Miura *et al.*, 2018 ▸): a U-Net model (Ronneberger *et al.*, 2015 ▸) to segment the liquid droplets followed by an Inception v3 architecture CNN (Szegedy *et al.*, 2016 ▸) to classify the experimental outcomes. The classifier network was pre-trained on a subset of the ImageNet database of images (Russakovsky *et al.*, 2015 ▸), which consists of 1.2 million images from 1000 mutually exclusive classes. Pre-training in this fashion results in a model that has already learnt a feature representation for extracting information from images. The weights from this network are then fine-tuned to perform a new classification task; the advantage of this strategy over training a model with randomized weights is fewer training epochs are required and there is less chance of overfitting the model when the training data set is small. The data set used in this particular study was a small subset of the MARCO data set (150–450 images for the U-Net and 99 images for the Inception v3 network). The authors concluded that using their two-CNN approach was superior to using one classifier network; however, the final precision and recall metrics for classifying the images are not given.

In another study, an EfficientNet CNN architecture (Tan & Le, 2019 ▸) was trained on the full MARCO data set (Edwards & Dinc, 2020 ▸). In this case, the same validation set was chosen as used by Bruno *et al.* (2018 ▸), but a separate test set of images was also created, comprising 10% of the original training set. The images were classified into the same four categories as MARCO. On the validation set they reported a precision of 0.87, a recall of 0.87 and an *F*1 score of 0.87 for the ‘Crystals’ class. On their test set they reported a precision of 0.95, a recall of 0.97 and an *F*1 metric of 0.96 for ‘Crystals’. This finding is somewhat unexpected, given that these data originally came from the same pool of images as the training and validation sets. For comparison, the authors then used the original MARCO model to classify the same test set (which had formed part of the training set for this particular model), resulting in a precision of 0.94, a recall of 0.91 and an *F*1 of 0.92 for ‘Crystals’; these metrics correspond to the published results in Bruno *et al.* (2018 ▸). The rationale behind utilizing this test set for comparisons rather than the original validation set is unclear.

As part of a study which carried out a further investigation into the structure of the MARCO data set, Rosa *et al.* (2023 ▸) trained a ResNet50 network on the data. They achieved a slight improvement in overall accuracy (94.63% versus the original MARCO score of 94.5%). The authors also found that supplementing the MARCO training data with local images (images from their own equipment in their institution) helped to improve the accuracy by as much as 6% when classifying these local images as opposed to using the MARCO data set alone. In addition, they examined the reasons behind the poorer performance of models trained on the MARCO data set alone when classifying images from sources that are not part of the original data. These reasons include data redundancy (duplicate images), the resolution of images included in the data, mislabelling of images, plate and imager types, and the inclusion of a small number of images from lipid cubic phase (LCP) experiments; these particular images can have a dramatically different appearance to images of standard sitting-drop vapour-diffusion experiments.

In their study, Milne *et al.* (2023 ▸) fine-tuned four different CNN architectures on their own data set of 16 317 images. The classifiers were pre-trained on ImageNet and were chosen to be trainable with more limited computational resources. The authors found that they achieved better performance by only using their own images and not supplementing them with those from the MARCO data set. Each model was found to have its own strengths and weaknesses at categorizing images into the eight categories that they had defined. For example, for images containing crystals, the Xception architecture (Chollet, 2017 ▸) was found to have the highest precision, at 0.96; however, the recall was 0.75, giving an *F*1 of 0.84. As a result of this, they opted to combine multiple classifiers into an ensemble of models, where majority voting was used, to achieve an accuracy comparable to that of MARCO. This was possible even when excluding the Xception network and just using the other three architectures, namely Inception v3, ResNet50 and DenseNet121 (Huang *et al.*, 2017 ▸).

Thielmann *et al.* (2023 ▸) trained four different CNN architectures on a data set generated by their own robotic imaging microscopes. These were AlexNet (Krizhevsky *et al.*, 2012 ▸), VGGNet (Simonyan & Zisserman, 2014 ▸), ResNet50 and SqueezeNet (Iandola *et al.*, 2016 ▸). They chose to adopt an approach similar to Miura *et al.* (2018 ▸), and first trained a U-Net network to detect the experimental droplets. After detection, the droplet region of interest (ROI) was further subdivided into image patches that were used to train a separate CNN model to classify the images into 12 classes. Their initial data-set size was 33 872 images and their process of subdividing the images led to >324 000 patches (the authors did not state the split between training and validation sets). In order to classify the images, it was again necessary to divide the image into patches and then classify each patch individually before arriving on a class for the parent image using a ranking system. They achieved the best overall success with the SqueezeNet architecture, although AlexNet gave the highest success for the ‘Crystals’ class.

### 1.2. Background on crystalline object detection in images

Despite many years of research effort dedicated to classifying experimental micrographs into categories, it is only more recently that the possibility of detecting the locations of protein crystals and/or drops within the images has been investigated. Object-detection techniques define a bounding box for the entity in the image and assign a class label, giving an approximate measure of size as well as the (*x*, *y*) coordinates. For more accurate sizing of entities in the image, instance segmentation can be used. Instance segmentation is the process in which individual objects in an image are separately identified before all of the pixels in the object are given a label. This allows the counting of, as well as the measurement of, crystals from images to give size distributions.

Crystal object detection and instance segmentation have been investigated most thoroughly in the context of batch experiments on proteins and small molecules in the field of chemical engineering. The use of reproducible procedures means that the resulting crystal morphology is often known, which allows model-based approaches to be used (Larsen *et al.*, 2006 ▸; Pons & Vivier, 1990 ▸). The size distributions measured by these techniques can be used to characterize the end products of industrial processes as well as to monitor the crystallization process over time.

#### 1.2.1. Object-detection metrics

To assess the performance of object-detection networks, the mean average precision (mAP) metric is most commonly used. The basis of the mAP metric is the precision and recall (as described in Section 1.1.1, equations 1 and 2) as used for classification tasks. However, in the context of object detection, where a bounding box is predicted for each object, the definition of what constitutes a correct classification is dependent on the degree of overlap between the predicted bounding box and the actual ‘ground-truth’ bounding box. This degree of overlap is measured using the intersection over union (IoU) metric, which is defined in equation (4). When considering instance segmentation, IoU is calculated in the same manner but with the intersection and union of the segmented areas as the equation terms.
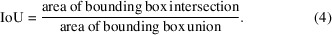


To calculate mAP, it is first necessary to plot a precision–recall curve for each class. This is a plot of how these two metrics change at different confidence thresholds for a classifier. The average precision, defined as the area under this curve, is then calculated for a range of IoU thresholds (although in some cases at just one IoU threshold). The mean average precision is the mean of these calculated average precision values when also averaged across all of the classes detected. In addition to defining the average precision by IoU threshold, evaluation can be split by object size to allow a comparison of performance on ‘small’ (<32 × 32 pixels) versus ‘large’ (>96 × 96 pixels) objects, for example.

Due to the complexities of mAP, to compare scores with one another it is important to be sure that the mAP has been calculated across the same range of IoU thresholds and also calculated on the same model output (bounding boxes versus segmentation masks). Although a higher mAP score means more accurate detection and localization of objects in images, there is no notion of what constitutes a good score. In all but one of the previous studies described here, no metrics were reported. In our study we report the mAP achieved so that it may serve as a benchmark for future work.

#### 1.2.2. Previous investigations of automated crystal detection

Gao *et al.* (2018 ▸) trained a network to perform object-instance segmentation of crystals of l-glutamic acid. The Mask R-CNN architecture that they used (He *et al.*, 2020 ▸) has a backbone network (often ResNet50 or similar) that extracts features as part of a feature-pyramid network (FPN). The levels within the FPN contain features at different resolutions, allowing the network to be able to detect objects at different scales in the image. The feature maps from the FPN are then passed to a region-proposal network (RPN), which predicts bounding-box proposals for regions of interest within the image. The underlying features are aligned with the region proposals before being passed on to parallel network components which output bounding-box coordinates, a class label for the object and a segmentation mask. The trained network was able to predict instance-segmentation masks for both the α and β crystal forms and allowed crystal size distributions for both populations to be determined from images. No performance metrics are given for this network, however.

Bischoff *et al.* (2022 ▸) trained a single-shot alignment object-orientated detection network (S^2^A OOD-Net; Han *et al.*, 2022 ▸) on simulated images of protein crystals in suspension. Rather than outputting bounding boxes of a fixed orientation, which was previously standard for object-detection networks, this network can orientate the boxes to achieve the best fit to crystals. The synthetic images were generated using ray-tracing algorithms, with the size and alignment of the crystals determined from a statistical distribution and the crystal shape chosen at random from a collection of 1851 models. This resulting training set contained 332 558 images. The model was evaluated by calculating size distributions of crystals in experimental images of *Lactobacillus kefir* alcohol dehydrogenase which had been grown in stirred-tank batch reactors. No metrics were calculated for precision of detection (such as mean adjusted precision).

In order to apply object-instance segmentation to sitting-drop protein crystallization micrographs, Qin *et al.* (2021 ▸) used a Mask R-CNN with a ResNet101 backbone network. This backbone had been pre-trained on the Common Objects in Context (COCO) data set (Lin *et al.*, 2014 ▸). The COCO data set contains 2.5 million labelled segmentation instances, and utilizing a model pre-trained on this data has the same advantages as utilizing classification networks pre-trained on the ImageNet data set. The images used for training their network were taken from the MARCO data set, but since these data are annotated solely with classification labels, instance-segmentation masks had to be created first. The masks were created manually on the images using a drawing tool named *Labelme* (Wada, 2023 ▸). The authors do not disclose how many images were annotated or the size of their training set, but they did report mAP scores of 0.47 (calculated on 100 images at an IoU threshold of 0.65) and 0.70 (calculated on ten images at an IoU threshold of 0.5). An improvement in the performance of their model was achieved when including an image pre-processing step, namely the *Contrast Limited Adaptive Histogram Equalization* (*CLAHE*) algorithm (Pizer *et al.*, 1987 ▸). A network trained on images which were not processed using *CLAHE* had lower mAP scores of 0.30 at an IoU threshold of 0.65 and of 0.67 at an IoU threshold of 0.5. This localized image enhancement potentially allows crystals in the shadow areas at the edges of drops to be discerned more easily and could also reduce reflection artefacts from the surface of the drops.

## 2. Justification for the creation of new, in-house, tools

To enable further automation of the VMXi robotic *in situ* macromolecular crystallography beamline at DLS, automatic categorization of experimental micrographs alongside detection of crystal positions for fully automated data collection was desired. The initial solution implemented for categorizing the images was to use the MARCO classifier network (Bruno *et al.*, 2018 ▸), as described in Section 1.1.2. The network was deployed in November 2019 and was tasked with classifying all images collected by the Formulatrix Rock Imager machines at the experimental facility. The predicted label probabilities and the resulting image labels were added into the ISPyB LIMS database and could then be viewed on a schematic plate overview in the SynchWeb interface by beamline users (shown in Supplementary Fig. S1). After an initial period of use, however, the feedback from those using the new feature was mixed, with mistrust that the images were being classified correctly. As a result of this, a small-scale experiment was then performed to evaluate the performance of the MARCO classifier on micrographs captured in-house. This work (on a limited set of 80 images) found that the *F*1 score for the MARCO network when categorizing images from VMXi as ‘Crystals’ (0.76) was slightly lower than reported for the same network on an independent test set of images (0.82; Bruno *et al.*, 2018 ▸). The main finding, however, was that the MARCO classifier tended to classify images as ‘Precipitate’ at the expense of other classes, with a recall of 1.0, a precision of 0.48 and a false-positive rate of 37%. This bias towards categorizing images as ‘Precipitate’ is understandable since ∼48% of the MARCO training data set is made up of images that were given this label and these images outnumber the images labelled as ‘Crystals’ by a ratio of ∼4:1. The methodology and more detailed results of this experiment are described in Section S1.

As was later confirmed by Rosa *et al.* (2023 ▸), we hypothesized that training with local images would be needed in order to achieve better classification performance since the images in the MARCO training data set come from different imagers and the experiments are set up in different plates to those used in our facility. Difficulties associated with adding training to the existing MARCO model, along with advances in the field and the need to perform object detection as well as image classification, led us to carry out experiments on training our own networks.

Automation of finding the (*x*, *y*) coordinates of any crystals present in the experimental images was desired for two different use cases. In the first, on the VMXi beamline, human intervention was required to locate each crystal and add it to a queue for data collection. At the time, this was performed using a web-based point-and-click interface in SynchWeb, which then added the coordinates to the ISPyB LIMS system. In the second case, as part of the XChem project at DLS, high-throughput fragment-based drug-discovery workflows required the acoustic dispensing of low-molecular-weight compounds into the experimental drops after crystals had grown. High levels of the solvent DMSO in the direct vicinity of the crystals can disrupt their structure and ability to diffract. Because of this, dispensing of the compounds is targeted within the drop but in a region away from the crystals to be mounted for data collection, thereby allowing the compound and associated DMSO to diffuse through the drop gradually towards the crystals of interest. At the time, this targeting step was performed via a manual point-and-click interface in a modified version of the *TeXRank* software (Ng *et al.*, 2014 ▸), but automation of this targeting was desired in order to increase the throughput of the screening pipeline.

## 3. Methods

The procedures used to collate the data and to train and evaluate the classification as well as the detection networks are described here.

### 3.1. Creating the CHiMP Classifier models

The data used to train the crystal-micrograph classification networks comprised two sets of images. The first, henceforth referred to as the VMXi Classification Data Set, is described in more detail in Section 3.1.1. The second set of images was obtained from the Machine Recognition of Crystallization Outcomes project (Bruno *et al.*, 2018 ▸) and is henceforth referred to as the MARCO Data Set (this data set was introduced in Section 1.1.2 and is further described in Section 3.1.2.

The initial work on classification networks used the VMXi Classification Data Set alone. This resulted in a ResNet50 classification model given the name CHiMP (Crystal Hits in My Plate) Classifier v1 (further details of the training of this classifier are given in Section 3.1.3). This network was put into production on the VMXi beamline for a period of three years. Although the feedback from use of this classifier was positive (to the extent that image classification by the MARCO classifier was eventually switched off), further work was started to investigate replacing the network. This was performed for two main reasons. Firstly, there was a software dependency on version 1 of the *fastai* package. The second version of the package had an API that was not backwards-compatible and development of the first version was stopped. Secondly, although the ResNet50 model weights could have been transferred to a purely PyTorch ResNet50 implementation, significant progress had been made in CNN architectures in the meantime. A more modern model architecture, namely ConvNeXt (Liu *et al.*, 2022 ▸), was chosen. As well as utilizing increased kernel sizes, ConvNeXt networks use some innovations commonly found in Vision Transformer (ViT) models, such as a reduced use of activation functions and normalization layers, and applies these modifications to a CNN to improve performance. The ConvNeXt-Tiny version of the model was chosen because it has a similar number of parameters to ResNet50. This model was trained on both the MARCO Data Set and the VMXi Classification Data Set, because it was hypothesized that this would lead to a model with improved classification performance when compared with one that had been fine-tuned on VMXi data alone. Once trained, this new model, named CHiMP Classifier v2, was put into production on the VMXi beamline in March 2023 (further details of the training of this classifier are given in Section 3.1.4).

#### 3.1.1. Curation of the VMXi Classification Data Set

Details of the collection and manual categorization of the images at the VMXi beamline experimental facility are given in Section S2.1.

At VMXi, a ten-class classification system is used to label the outcomes (see Table 1 ▸). The initial set of images that make up the VMXi data set were found by querying the ISPyB LIMS for images which had associated scores and that had been collected up to the query date (March 2020). The set of images was also further restricted to those from experiments belonging to beamline users who were not from commercial/industrial organizations in order to abide by intellectual property restrictions and agreements. This resulted in a set of 18 782 images. To remove redundancy in the data caused by the inclusion of similar images from multiple inspections of the same subwell, the data were grouped by subwell and the inspection numbers were analysed. The majority of the subwells only had one image with an associated score (11 167 images); these were included in the final data set. In the cases where subwells had images with scores for more than one inspection, the image and the associated label from the second of these inspections was added to the data set (2784 images). This resulted in a final data-set size of 13 951 images.

The labels assigned to the images were mapped from the ten in-house categories to a four-class system (as used by MARCO): ‘Crystals’, ‘Precipitate’, ‘Clear’ and ‘Other’. This mapping can be seen in Table 1 ▸. A breakdown of the number of images in each category at this stage can be found in Supplementary Table S2.

**Figure 1:**
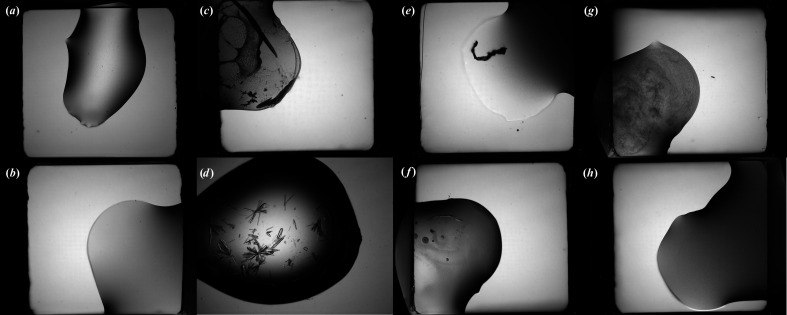
Examples from the VMXi Classification Data Set. Two images from each category were selected at random from the training data set. (*a*, *b*) Images from the ‘Clear’ class. (*c*, *d*) Images from the ‘Crystals’ class. (*e*, *f*) Images from the ‘Other’ class. (*g*, *h*) Images from the ‘Precipitate’ class.

This data set was used as the starting point for fine-tuning a ResNet50 network that had been initialized with weights from training on ImageNet. Further details of the training method can be found in Section 3.1.3. During the initial rounds of training, it became apparent that the training labels associated with the images were not always accurate, so a strategy to clean the image labels was implemented. Details of this method can be found in Section S2.2. A total of 904 labels were changed during this process (6.5% of those in the data set); an overview of the changes broken down by image class can be seen in Supplementary Table S2. After cleaning, the data were again split at random into a training set comprising 80% (11 161) of the images and a validation set of 20% (2790 images). The final number of images in each category can be seen in Table 2 ▸. Example images from each class can be seen in Fig. 1 ▸. The data set is available to download from https://doi.org/10.5281/zenodo.11097395.

#### 3.1.2. The MARCO data set

As described in Section 1.1.2, the Machine Recognition of Crystallization Outcomes (MARCO) initiative (Bruno *et al.*, 2018 ▸) collated 493 214 labelled images from five different organizations to create a data set for training their CNN classification model. Ambiguous labels were cleaned in a similar fashion to that described for the VMXi Classification Data Set in Section S2.2, with the top 5% of images (ranked by classification loss) revisited by expert crystallographers. In this 5% of the images, 42.6% were relabelled, which highlights the ‘label noise’ present in the data set.

**Figure 2:**
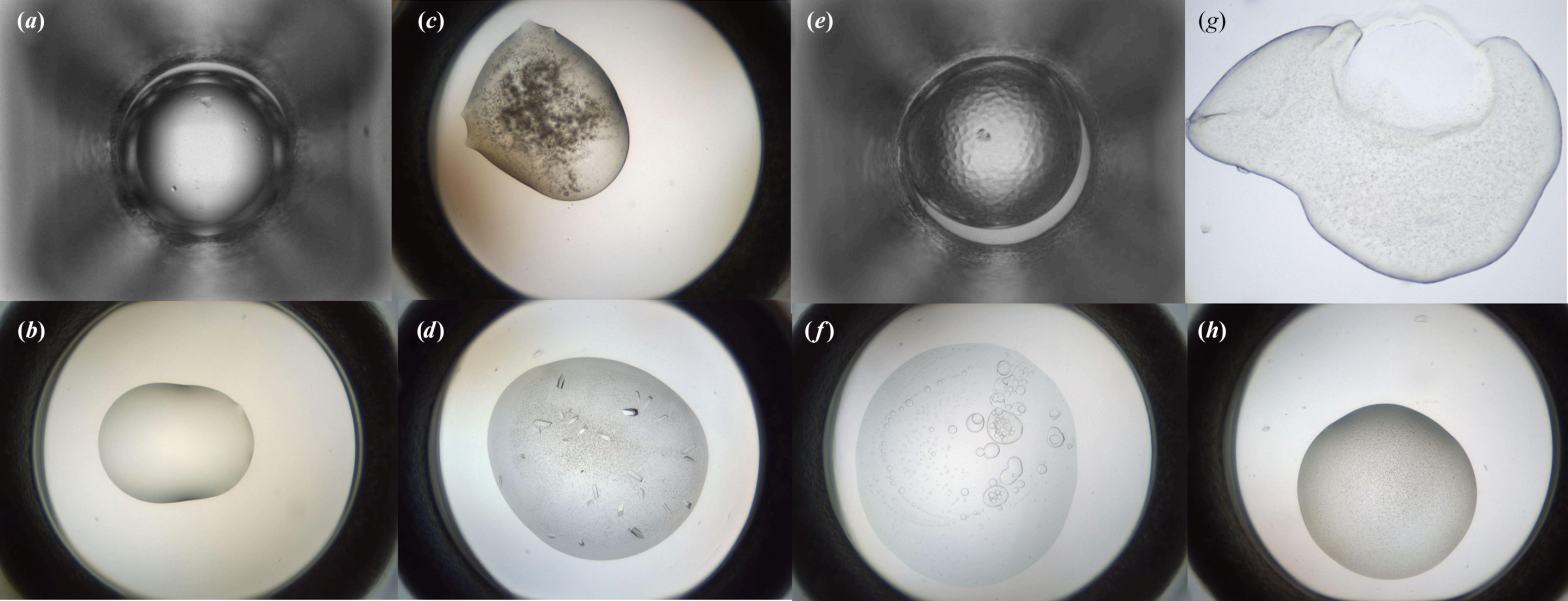
Examples from the MARCO Data Set (Bruno *et al.*, 2018 ▸). Two images from each category were selected at random from the training data set. (*a*, *b*) Images from the ‘Clear’ class. (*c*, *d*) Images from the ‘Crystals’ class. (*e*, *f*) Images from the ‘Other’ class. (*g*, *h*) Images from the ‘Precipitate’ class.

After cleaning, the authors divided the data into training and validation sets comprising ∼90% and ∼10% of the images, respectively. In their paper, Bruno *et al.* (2018 ▸) give a table summarizing the breakdown of image classes in the MARCO Data Set (with 442 930 training and 50 284 validation images); however, the data set that they used for training is described as having 415 990 training images and 47 062 validation images. The numbers of images in the data set that they released publicly differs slightly again. A summary of the number of images in each category in this publicly released set is given in Table 3 ▸, since this is the one used in our investigations. A detailed exploration of the MARCO Data Set can be found in Rosa *et al.* (2023 ▸). Example images from each class can be seen in Fig. 2 ▸.

#### 3.1.3. Training of CHiMP Classifier v1

This initial work on classification networks used the *fastai* Python library (Howard & Gugger, 2020 ▸), which is a high-level interface to the PyTorch machine-learning framework (Paszke *et al.*, 2019 ▸).

A ResNet50 convolutional neural network (CNN; He *et al.*, 2016 ▸) that had been initialized with weights from a model trained on the ImageNet database (Russakovsky *et al.*, 2015 ▸) was the starting point for our experiments. This model was fine-tuned on the VMXi Classification Data Set (described in Section 3.1.1) in a number of phases with increasing image dimensions. To prevent overfitting to the training data (to make up for the relatively small number of images available), during each phase of training the training set of 11 161 images was augmented using a randomized range of flips, rotations, warping and zooming as well as brightness and contrast adjustments. Before passing the images through the network, they were reshaped (or ‘squished’) to a square, rather than cropped; this was performed to avoid objects at the edge of the image, such as crystals, being excluded. In addition, the image histograms were normalized to match those used by the ImageNet database. In order to make up for the label imbalance in the VMXi Classification Data Set (where images labelled as ‘Crystals’ make up ∼60% of images, whereas those in the ‘Other’ class make up just 6.6%), an oversampling method was used. This method sampled the image classes in the data set in inverse proportion to their frequency, thereby counteracting this imbalance. In total, the model was trained for 31 epochs with a batch size of 64; the final model had a cross-entropy loss of 0.115 for the training set and 0.364 for the validation set. Training was carried out on an NVIDIA Tesla P100 GPU with 16 GB of VRAM and took around 6.5 h of computation time. Further details of the training strategy can be found in Section S2.3. Metrics for the validation set of images from the VMXi Classification Data Set are given in Section 4.1.1. Evaluation of the performance of this model on an independent test set of images from the VMXi facility is given in Section 4.1.2.

**Figure 3:**
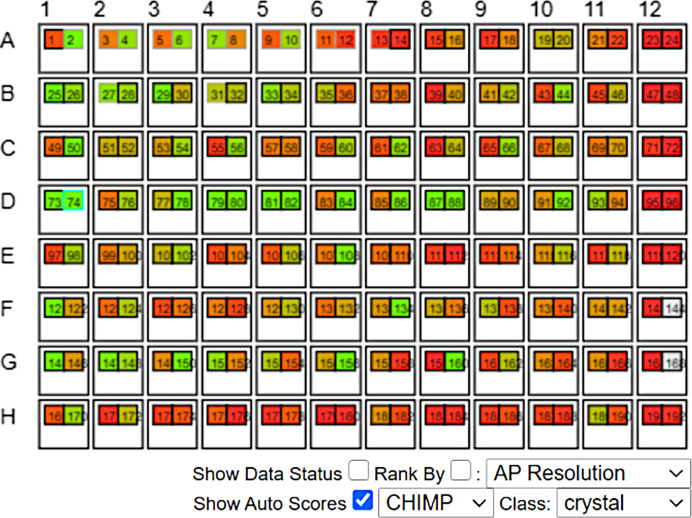
Schematic overview of classification outputs from the CHiMP Classifier v1 CNN, as displayed in the SynchWeb interface for users of the VMXi beamline at Diamond Light Source. The 96-plate wells are represented as larger boxes, with the two subwells containing crystallization droplets shown as smaller boxes within. Each subwell is assigned a colour on a gradient from red to green that represents the probability of the associated image being assigned to the class ‘Crystals’, with green representing a high probability.

The network was deployed alongside MARCO to classify all experimental images that were produced by the imagers on the VMXi beamline. The classification labels were inserted into the SPyB LIMS and the results were displayed on a plate-overview schematic in the SynchWeb interface (shown in Fig. 3 ▸).

#### 3.1.4. Training of CHiMP Classifier v2

In a similar manner to the work on the first version of the classifier, the starting point for training was a model initialized with weights from ImageNet pre-training. This model was then fine-tuned using custom PyTorch training routines. Unlike version 1 of the CHiMP classifier, in this case the first stage of training was performed using the MARCO Data Set (described in Sections 1.1.2 and 3.1.2), and the final training stages used the VMXi Classification Data Set (described in Section 3.1.1).

The pre-trained ConvNeXt-Tiny network was obtained from the PyTorch Image Models library (Wightman, 2019 ▸). The standard one-layer classification head of the CNN was replaced with a two-layer fully connected network with batch normalization layers and a dropout of 0.4 on the last layer, rather than using a standard, single fully connected layer. An initial training epoch was carried out with all convolutional parameters frozen, training only this classification head to start with.

Several training phases were implemented, with increasing image dimensions in each phase, before final training epochs on images with dimensions of 512 × 512 pixels. Images were resized to the required input dimensions by rescaling followed by padding to ensure that objects such as crystals at the edge of the image were not removed by cropping. Image augmentations that were used on the training set included random flips and rotations, optical distortions, brightness and contrast alterations, blurring and contrast-limited adaptive histogram equalization (*CLAHE*). The imbalance in the number of images in each category in the data set was counteracted by sampling the data in inverse proportion to the frequency of each category. After each training epoch, the model was saved and used as the model for the next epoch only if the overall classification accuracy on the validation set had improved in comparison to the previous best model. To prevent over-fitting, an early stopping safeguard was used, which terminated model training if the validation accuracy stopped improving after a predefined number of epochs. As for version 1 of the classifier, the optimizer for model parameters was AdamW (Loshchilov & Hutter, 2017 ▸), and cross-entropy loss was used. The learning rate was again cycled during training on a ‘1cycle’ schedule (Smith & Topin, 2019 ▸).

Further details of the training phases can be found in Section S2.4. After 12 epochs of fine-tuning on the MARCO Data Set, the cross-entropy loss for the training set was 0.201 and that for the validation set was 0.214. After a further 24 epochs of fine-tuning on the VMXi Classification Data Set, the final cross-entropy training loss was 0.421 and the final validation loss was 0.522. Metrics on the validation set of images from the VMXi Classification Data Set are given in Section 4.1.1. Evaluation of the performance of this model on an independent test set of images from the VMXi facility is given in Section 4.1.2.

This new model, named CHiMP Classifier v2, was put into production on the VMXi beamline in March 2023.

#### 3.1.5. Creation of test sets of images to evaluate model performance

A test set of images from the robotic imaging microscopes on the VMXi beamline was created and scored by a panel of experts to evaluate the classification performance of the networks. At the time of creation of this test set (August 2022), the CHiMP Classifier v1 network had been categorizing all images collected by the imagers for more than two years, with the results inserted into the ISPyB LIMS. The LIMS was queried for scored image labels and returned data for around 780 000 classified images. These classifications were first grouped by inspection number and plate subwell before limiting the set to those with two inspections or more in order to exclude images taken of unusual experiments that were not being monitored over time. The remaining set consisted of 751 540 images from 69 542 subwells.

The groups of images for each subwell represent time courses over which the experiments were monitored. To prevent multiple similar images from the same subwell appearing in the data set, one image was selected at random from each of these time courses in the range of inspection 2 to inspection 7. This population of 69 542 images with associated classification labels was then sampled to create a final data set of 1000 images with 250 members from each class of the four-category system used by CHiMP Classifier v1 (‘Crystals’, ‘Precipitate’, ‘Clear’ and ‘Other’). This balancing was performed to ensure that the data set included sufficient numbers of images from each class to be able to draw robust conclusions.

A panel of three experts viewed all images independently and assigned one of the four class labels to each image. The experts did not always agree with each other (described further in Section 4.1.2). To accommodate this ambiguity in labelling, two subsets of the data set were created to assess the models against. The first, named the unambiguous test set, consisted of the 632 images where all three experts agreed on a label. The second, named the mostly unambiguous test set, consisted of 949 images where at least two experts agreed on the label. The number of images assigned to each category for each of these data sets can be found in Table 4 ▸.

### 3.2. Training Mask R-CNN networks to detect objects in experimental micrographs

Initial experiments used gradient-weighted class-activation mapping (Grad-CAM; Selvaraju *et al.*, 2017 ▸) to locate regions of the image where CHiMP Classifier v1 was being activated when predicting the ‘Crystals’ class. However, the low resolution of the output maps (16 × 16 pixels) meant that this technique was not able to provide a positional accuracy that was high enough for our purposes. An alternative approach of training a CNN, such as a U-Net, to perform segmentation of the crystals from the rest of the image was also considered. The disadvantage of this approach is that a semantic segmentation technique such as this would divide the image into two classes, crystals and background, but would not separate out individual instances. Detecting individual crystal instances would be preferable, allowing those that are overlapping or growing in conjoined inflorescences to be detected and targeted individually. For these reasons, the use of an object-detection CNN was investigated, in this case a Mask R-CNN architecture (described in Section 1.2.2), which has the advantage of also providing instance segmentation alongside bounding-box suggestions, providing the potential to allow more accurate measurement of crystal dimensions and calculation of the centre of mass for targeting.

#### 3.2.1. Image selection for detection networks

To obtain images to train the detection network for the VMXi beamline, a random selection of 1000 experimental images from different plate subwells was first taken from the set of all images collected up to this point (March 2021), excluding those linked to industrial experiments. These images were then classified automatically using the CHiMP Classifier v1 network before a data set of 250 images was sampled from this initial selection. The sampling was performed with 50% (125 images) coming from those images labelled as ‘Crystals’, ∼25% (62 images) labelled as ‘Precipitate’, ∼20% (51 images) labelled as ‘Clear’ and ∼5% (12 images) labelled as ‘Other’. Since each image would need to be annotated manually, the size of the training data set that could be created would be limited; therefore, this split was chosen to prioritize images with crystals present whilst also including those with a range of different outcomes for the network to learn from. These images were then inspected manually and similar images were discarded, resulting in a final data-set size of 237 images. These images were then scaled down by a factor of two to 1688 × 1352 pixels before having their histograms adjusted by the *CLAHE* algorithm (Pizer *et al.*, 1987 ▸) using the OpenCV library (Bradski, 2000 ▸) with grid size of 12. This was performed to enhance the visibility of crystals in shadow areas and reduce the glare from reflections on droplets.

To obtain images to train the detection network for the XChem Fragment-Based Drug Discovery facility, the integrated database of the Rock Imager 1000 robotic imaging system (Formulatrix, USA) located at the Crystallisation Facility at Harwell was queried (October 2022). The images were then grouped by plate subwell and a selection of 1000 images were chosen at random from this grouped set. These images were then viewed individually and reduced to a set of 350 images that showed a diversity of experimental outcomes, plate types and crystal forms. These images, with dimensions of 1024 × 1224 pixels, then had their histograms adjusted using the *CLAHE* algorithm in the same manner as for the VMXi image data set.

#### 3.2.2. Creating image annotations for detection networks

When training a Mask R-CNN model, three sets of label data are needed for every object contained within the image, alongside the image files themselves. These are (i) object masks (the outline of each object), (ii) object class labels and (iii) object bounding-box coordinates (the coordinates of the vertices of a rectangular selection that encloses each object). Manually generating this information for each image can be arduous depending on the image content, for example in the case where there are hundreds of protein microcrystals in a droplet. In order to generate enough high-quality image annotations to train a network, the task of annotating was shared amongst experts at DLS using a custom project on the Zooniverse web platform (https://www.zooniverse.org/; Simpson *et al.*, 2014 ▸). A Zooniverse workflow was created with drawing tools that could be used to create polygonal masks on experimental droplets and on the crystals observed in the images. The set of images to be annotated was uploaded to the platform and each expert annotator was given access to the project. Supplementary Fig. S2 shows an example of using this workflow. The platform randomly selects images from the pool of unannotated images that remain and displays them to the expert alongside the web-based annotation tools. Once completed, the annotations can be downloaded from the platform in the form of a comma-separated values (CSV) file with the data contained in JavaScript Object Notation format (JSON) strings within this file. Methods were written in Python to extract the data from these strings and convert them into labels, masks and bounding boxes suitable for training a Mask R-CNN network (available at https://doi.org/10.5281/zenodo.11244711).

#### 3.2.3. Training the VMXi CHiMP Detector network

The 237 images and associated annotations comprising the VMXi Detector Data Set were split into training and validation sets, with 190 images (∼80%) in the training set and 47 images in the validation set. Since only the positions of the crystals were required, only the labels for the crystals were used in training; this gave better metrics in initial experiments compared with including labels for drops at the same time (results not shown). Because of the small size of the training data set, images (along with the associated object masks and bounding boxes) were augmented using random flips and rotations as well as random blurring, sharpening and contrast and brightness adjustments. The model was initialized with weights from a model pre-trained on the COCO data set. Training was performed for 20 epochs. The learning rate was cycled during training using a ‘1cycle’ policy (Smith & Topin, 2019 ▸) with an average learning rate of 1 × 10^−4^. The optimizer used was AdamW (Loshchilov & Hutter, 2017 ▸). The different components of the Mask R-CNN model use different loss functions (for example mask loss, bounding-box loss and classification loss), which are combined into one value named multi-task loss for monitoring during training. Training was performed with a batch size of 4 on an NVIDIA P100 GPU with 16 GB of VRAM and took one hour and ten minutes to complete. The final multi-task training loss was 0.7749 and the final multi-task validation loss was 0.8335. This model was named the VMXi CHiMP Detector network and was deployed on the VMXi beamline in December 2021. Crystal targeting coordinates are calculated by taking the centroid (centre of mass) of the segmentation masks for each crystal detected. These coordinates are then inserted into the ISPyB LIMS database. The SynchWeb interface for the VMXi facility was altered to display these points and to allow them to be queued for data collection.

#### 3.2.4. Training the XChem CHiMP Detector network

The training of a Mask R-CNN for use on images from the XChem FBDD experiments took place approximately one year after the training of the VMXi CHiMP Detector network. Because of this, the strategy for training was different. In this case, the starting model was a Mask R-CNN that had already been fine-tuned for 20 epochs on both the crystal and drop annotations from the 237 images in the VMXi Detector Data Set. This model was then trained further on the 350 images and associated annotations that make up the XChem Detector Data Set. The data were split randomly, with 280 images (80%) in the training set and 70 images in the validation set. The training was performed in the same fashion as for the VMXi CHiMP Detector, with image augmentations including random flips and rotations as well as random blurring, sharpening and contrast and brightness adjustments. Once again, a ‘1cycle’ policy (Smith & Topin, 2019 ▸) was used along with an AdamW optimizer (Loshchilov & Hutter, 2017 ▸). Training was performed for 50 epochs with an average learning rate of 5.2 × 10^−4^. The batch size used was 4 and the training was performed on an NVIDIA V100S GPU with 64 GB of VRAM and took 1.25 h to complete. The final multi-task training loss was 0.7069 and the final multi-task validation loss was 0.6962. This model was named the XChem CHiMP Detector network.

#### 3.2.5. Using the XChem CHiMP Detector network to calculate compound-dispensing positions

Using outputs from the Mask R-CNN trained on images from the XChem project, an algorithm was developed to determine a candidate position in the experimental drops for acoustically dispensing compound fragments. The network creates segmentation masks for drops detected in addition to a mask for every crystal detected. The ideal position for dispensing compound is (i) away from the edge of the drop yet also (ii) away from where the crystals are located. A position that meets these two criteria was calculated as follows.

**Figure 4:**
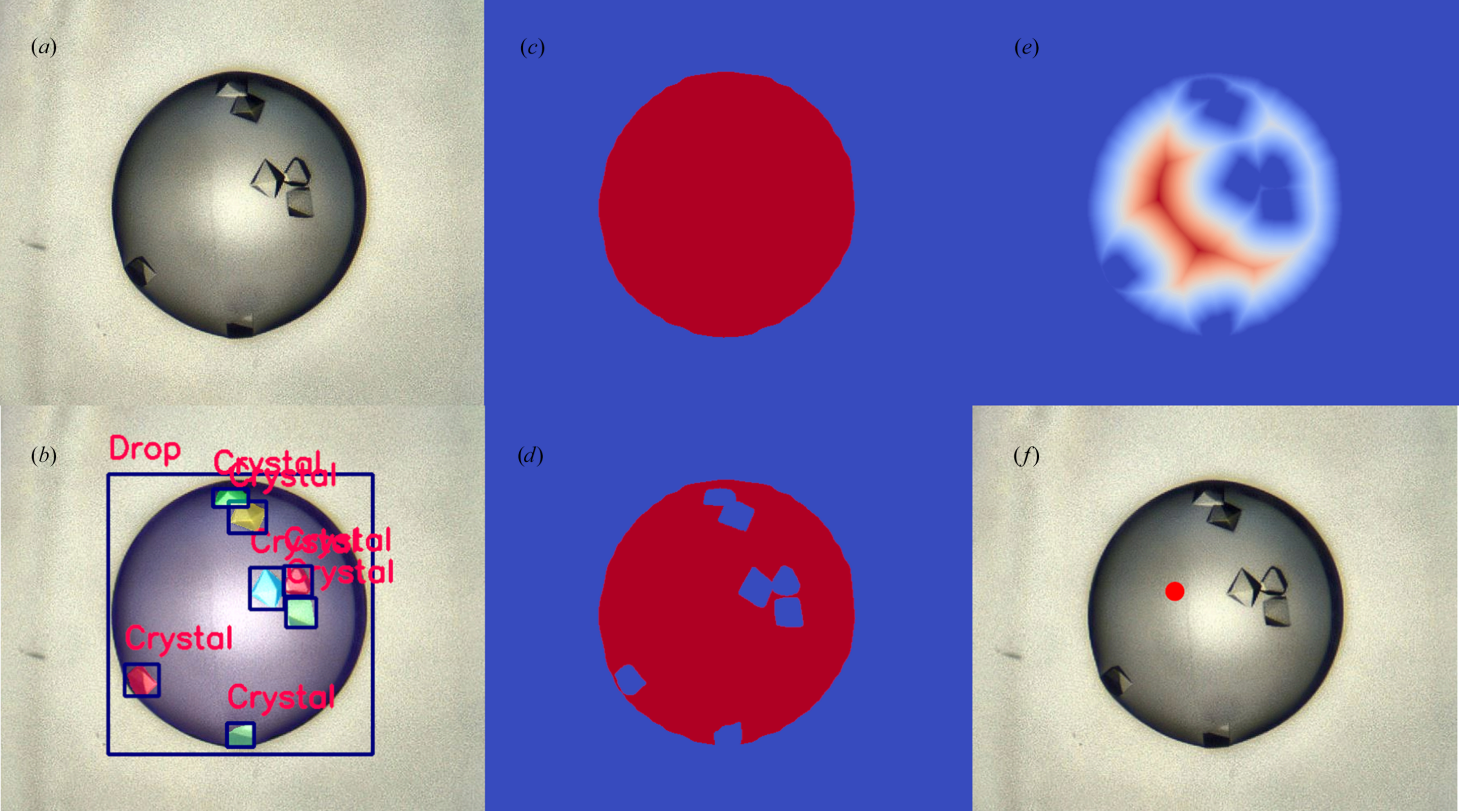
Using the output from the XChem CHiMP Detector Mask R-CNN model to determine coordinates to dispense compound for fragment soaking. For the mask images, low pixel values are depicted in blue and high pixel values are depicted in red. (*a*) Original micrograph of a crystallization drop. (*b*) The output bounding boxes, labels and masks from the XChem CHiMP Detector network. Bounding boxes are dark blue and associated object labels are in red. Instance-segmentation mask colours are randomized. (*c*) The predicted mask for the drop. (*d*) The mask for the drop minus the union of the predicted crystal masks. (*e*) The Euclidean distance transform of the mask in image (*d*); red pixels are those farthest from an edge. (*f*) The original image showing the coordinates determined by taking the maximum pixel value from image (*e*) as a red dot; this is the suggested dispensing coordinates.

The segmentation mask for every crystal detected was subtracted from the drop mask, resulting in a single mask which encodes the shape of the drop minus the shape of the union of all crystals in that drop. This binary mask was then processed using an exact Euclidean distance transform. This transform replaces each pixel in the mask with a value denoting the Euclidean distance to the nearest pixel outside the mask (for further details of this transform, see Strutz, 2021 ▸). After the transform, the pixel with the highest value is that which is furthest away from the edge of both the drop and the crystals. The coordinates of this pixel are used as the suggested coordinates for dispensing compound. Fig. 4 ▸ shows an example of this process.

## 4. Results

### 4.1. Classification-network performance

After training, the performance of the classification networks was assessed using metrics calculated on the validation data as well as on the independent test sets.

#### 4.1.1. Performance on the VMXi classification validation set

On the validation set of the VMXi Classification Data Set, CHiMP Classifier v1 achieved a precision, recall and *F*1 score for the ‘Crystals’ class of 0.95, 0.92 and 0.94, respectively. For version 2 of the classification network, the precision, recall and *F*1 metrics for the ‘Crystals’ class were 0.97, 0.88 and 0.93, respectively, on the same data set. The metrics for the other image classes can be found in Supplementary Tables S5 and S6.

#### 4.1.2. Performance on the MARCO validation set

On the validation set of the MARCO Data Set, CHiMP Classifier v1 achieved a precision, recall and *F*1 score for the ‘Crystals’ class of 0.38, 0.62 and 0.48, respectively. For version 2 of the classification network, the precision, recall and *F*1 metrics for the ‘Crystals’ class were 0.71, 0.83 and 0.76, respectively, on the same data set. The metrics for the other image classes can be found in Supplementary Tables S7 and S8.

#### 4.1.3. Performance on the VMXi classification test sets

As described in Section 3.1.5, a set of 1000 images from the robotic imaging microscope at VMXi was collated. All of the images were scored by a panel of three experts, and two sets were created based upon either unanimous agreement between the experts (the unambiguous set) or majority agreement (the mostly unambiguous set). The pairwise agreement between classifications given by the experts ranged from 72.9% to 75.4%, showing that the experimental outcomes can be ambiguous to human scorers. All three experts agreed on 63.2% of the images. One of the experts was tasked with scoring the test set of images twice with a period of six months between sessions and was found to agree with themself 83.0% of the time.

The per-class precision, recall and *F*1 metrics for classifying both sets of images were calculated for CHiMP Classifier v1, CHiMP Classifier v2 and the MARCO classifier. Table 5 ▸ summarizes the per-class *F*1 metrics for each model on both of these data sets.

The precision and recall values for these models can be found in Supplementary Table S9, alongside metrics for a number of other models trained using different strategies or with a different architecture for comparison. Other models trained include ConvNeXt-Tiny models trained either solely on the MARCO data set or solely on the VMXi Classification Data Set plus a ResNet50 model trained using the same strategy as for the final CHiMP Classifier v2 ConvNeXt model.

### 4.2. Detection-network performance

As described previously (Section 1.2.1), the most widely used metric to describe the performance of object-detection networks is mean average precision (mAP). This metric was calculated for various IoU thresholds on the validation set of images for the both the VMXi CHiMP Detector and XChem CHiMP Detector networks.

#### 4.2.1. VMXi CHiMP Detector

The VMXi CHiMP Detector network was evaluated on the 47 images in the validation set of the VMXi Detector Data Set and achieved a mAP of 0.31 for bounding boxes on the ‘Crystals’ class when using the standard COCO IoU threshold ranges from 0.5 to 0.95 (with a step size of 0.05). Full mAP results are shown in Table 6 ▸. The detector network was also evaluated on the VMXi classification test sets (described in Section 3.1.5). The results of this analysis can be seen in Table 7 ▸.

**Figure 5:**
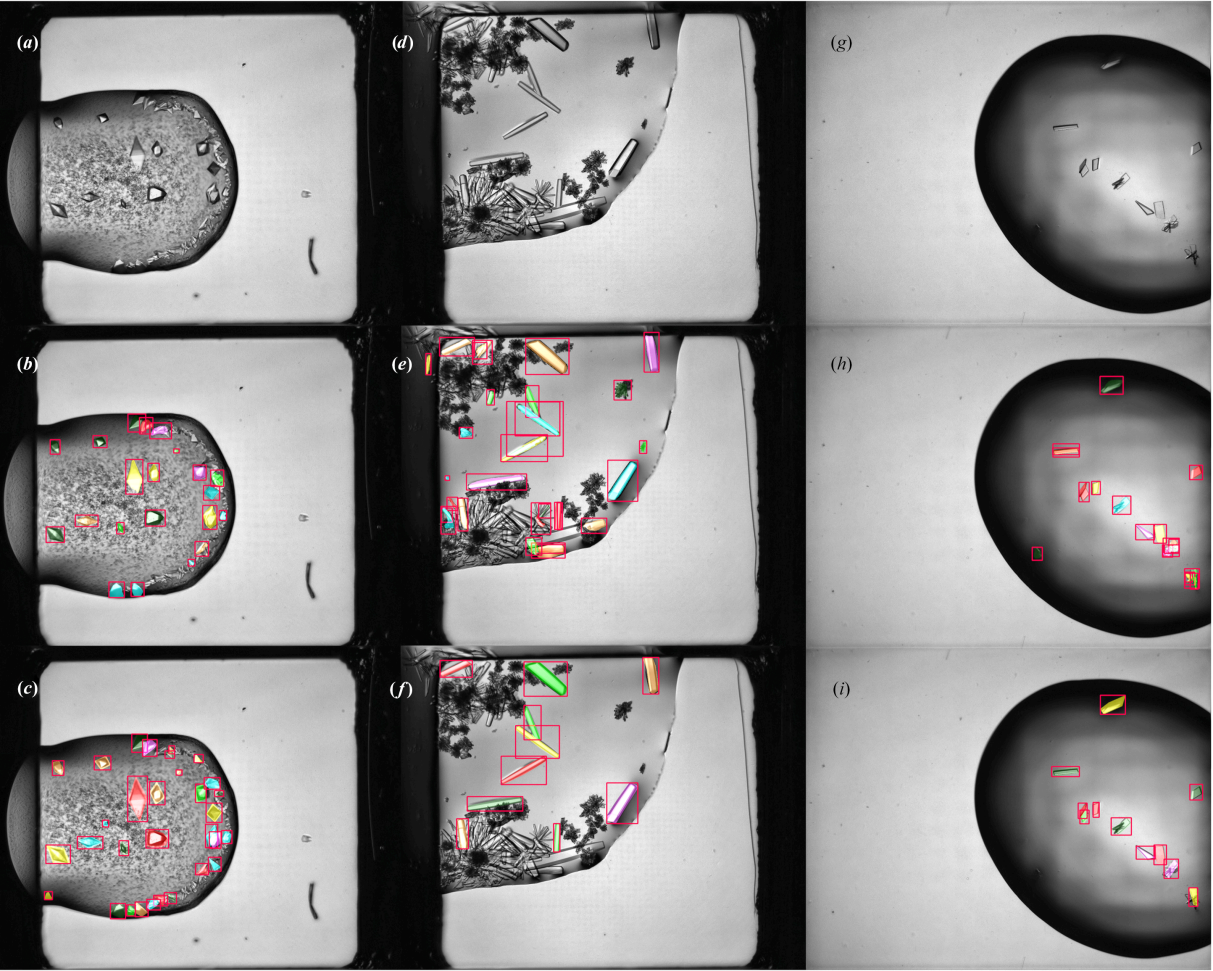
Output from the VMXi CHiMP Detector Mask R-CNN model on members of the validation set from the VMXi Detector Data Set. Red bounding boxes denote objects in the ‘Crystals’ class. Instance-segmentation mask colours are randomized. (*a*, *d*, *g*) Original micrographs of crystallization drops. (*b*, *e*, *h*) The corresponding bounding boxes and masks as predicted by the VMXi CHiMP Detector network. Objects were predicted with a probability of ≥0.6. (*c*, *f*, *i*) The ground-truth bounding boxes and masks from the VMXi Detector Data Set as annotated by an expert crystallographer.

Example outputs from the detector network can be seen in Fig. 5 ▸. The display of VMXi CHiMP Detector results in SynchWeb, alongside the plate schematic with a classification overview giving CHiMP Classifier v2 results, can be seen in Supplementary Fig. S3. An additional view of the display of detection results on their own can be seen in Supplementary Fig. S4.

#### 4.2.2. XChem CHiMP Detector

**Figure 6:**
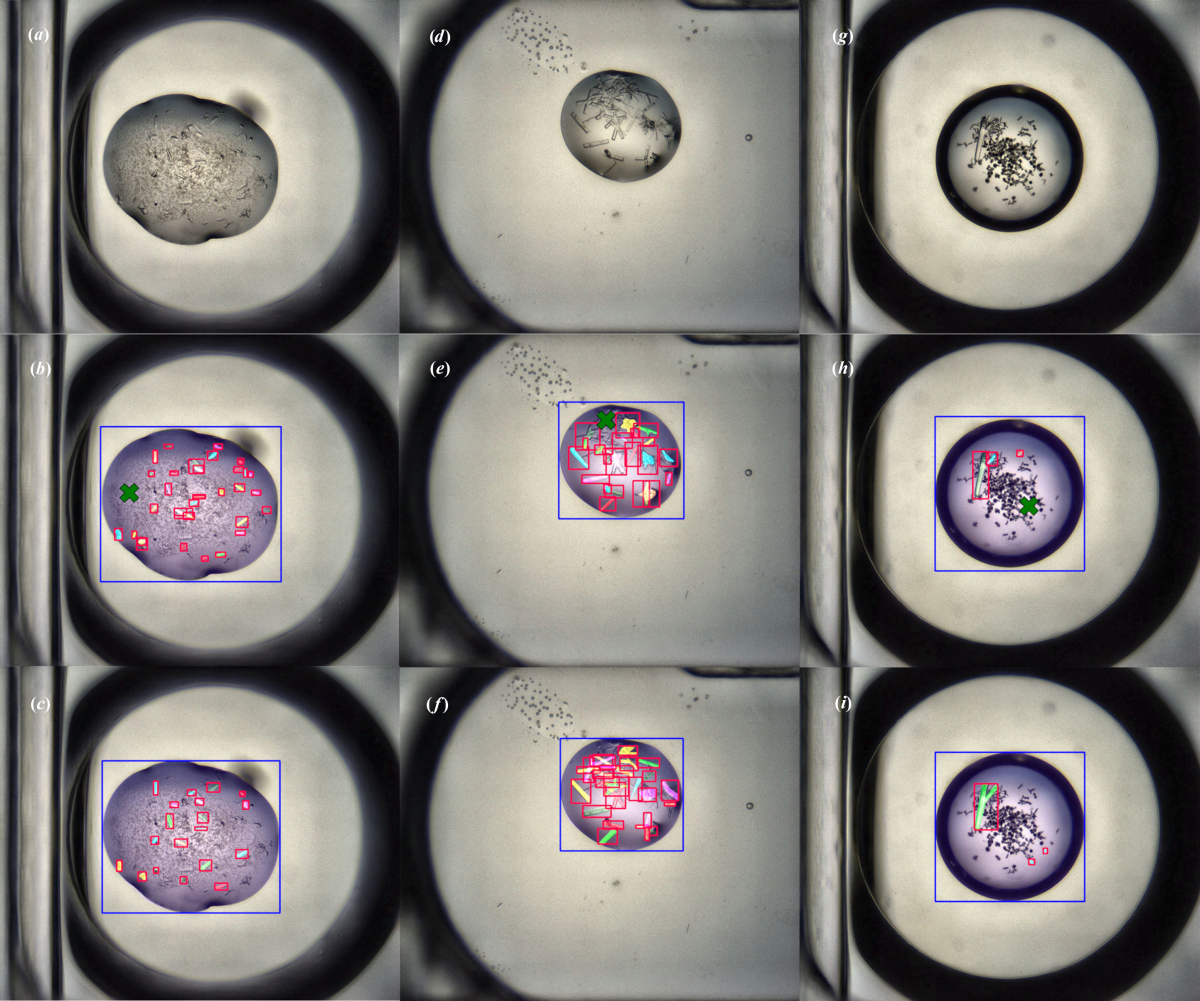
Output from the XChem CHiMP Detector Mask R-CNN model on members of the validation set from the XChem Detector Data Set. Blue bounding boxes denote objects in the ‘Drops’ class and red bounding boxes denote objects in the ‘Crystals’ class. Instance-segmentation mask colours are randomized. Green crosses denote candidate positions to dispense compound for fragment soaking (calculated via a distance transform). (*a*, *d*, *g*) Original micrographs of crystallization drops. (*b*, *e*, *h*) The corresponding bounding boxes and masks as predicted by the XChem CHiMP Detector network. Objects were predicted with a probability of ≥0.6. Candidate dispensing positions were calculated from output masks. (*c*, *f*, *i*) The ground-truth bounding boxes and masks from the XChem Detector Data Set as annotated by an expert crystallographer.

The XChem CHiMP Detector network was evaluated on the 70 images in the validation set of the VMXi Detector Data Set and achieved a mAP of 0.380 for bounding boxes averaged over the ‘Crystals’ class and the ‘Drops’ class when using the standard COCO IoU threshold ranges from 0.5 to 0.95 (with a step size of 0.05). Full mAP results are shown in Table 8 ▸. The detector network was also evaluated on the VMXi classification test sets (described in Section 3.1.5). The results of this analysis can be seen in Table 9 ▸. Example outputs of the network on members of the XChem Detector validation set can be seen in Fig. 6 ▸.

## 5. Discussion

In this study, deep-learning networks were fine-tuned, either to classify images into categories of experimental outcome or to detect and segment objects in the images to allow the targeting of locations for data collection or compound dispensing. This was performed to deliver increased automation for the scientists who normally must undertake these repetitive and time-consuming tasks.

### 5.1. On the classification of experimental outcomes

In order to train a network to classify the experimental outcomes, a robust set of data is needed for training. This study has relied on two data sets for image classification: the VMXi Classification Data Set (described in Section 3.1.1) and the MARCO Data Set (Bruno *et al.*, 2018 ▸; described in Section 3.1.2). These data sets both contain labels created by experts but this, in itself, does not mean that these labels are robust. Firstly, as mentioned previously, the categories for classification differ between different institutions, so when images from multiple sources are collated (as in the case of the MARCO Data Set) the labels need to be standardized into a system that may not adequately describe the complexity of the outcomes, thereby leading to ambiguity in the categorization. This oversimplification is compounded by the fact that each image is only given one label when, in fact, an image often contains multiple outcomes. An image that contains both precipitate and microcrystals will only be given the label ‘Crystals’. Multi-label classification is commonly used in other fields of study; in satellite remote sensing, for example, where one image may contain forest, savanna and a river, it would be given all three labels. Although studies do exist where a larger number of categories have been used to capture the outcome with more granularity, multi-label classification data sets do not exist for protein crystallization outcomes. This is largely due to historical precedent, where the labelling of images is made as simple as possible to ease the burden on annotators. To further ease the burden, annotating images is often optional, which then leads to unbalanced data since the crystallographer is most likely to label crystalline outcomes and to provide no label at all to other outcomes.

The ambiguity between labels assigned by domain experts is highlighted by the process used in this study in order to obtain a ‘ground-truth’ test set of images for model evaluation (described in Section 4.1.2). When categorizing 1000 images, three experts agreed only 63% of the time, the maximum pairwise agreement was 75% and the same expert agreed with themself 83% of the time when reassigning labels to the same images six months later. The fact that three experts with many years of combined experience could not agree on a label for 51 of the images also goes to show that the boundaries between categories are flexible. It is this ambiguity that makes the task nontrivial, despite the fact that image classification by CNNs is seen as something that can now be routinely performed with super-human accuracy in other domains (He *et al.*, 2015 ▸). The catch-all class of ‘Other’, in particular, seems to be ambiguous. When cleaning the labels for the VMXi data set (described in Section S2.2), more than 15% of the images in the ‘Other’ category had their label changed by an expert reviewer. For the models, correct classification of this class involves capturing features in the images which encompass a wide diversity of outcomes and therefore is also the most problematic. Of all the models trained, CHiMP Classifier v1 achieved the best performance on the ‘Other’ class, with an *F*1 score of just 0.47 on the unambiguous test set and 0.45 on the mostly unambiguous test set. The difficulties of accurately predicting this category are apparent in the data presented in Table 5 ▸ and Supplementary Tables S5, S6 and S9.

When compared with the MARCO classifier, which has been seen as the most robust model for classifying images of crystallization droplets in recent times, in this study we were able to achieve overall higher *F*1 metrics (averaged across all classes) on both our unambiguous and mostly unambiguous test sets of images from the VMXi beamline. This was achieved by fine-tuning a pre-trained classifier either solely on local images (for CHiMP Classifier v1) or with a combination of the MARCO Data Set and local images (for CHiMP Classifier v2); these results are shown in Section 4.1.2 and Table 5 ▸. When considering the ‘Crystals’ class alone, however, only the CHiMP Classifier v2 model (*F*1 of 0.83 on the unambiguous test set and 0.73 on the mostly unambiguous test set) was able to surpass the MARCO model (*F*1 of 0.78 on the unambiguous test set and 0.69 on the mostly unambiguous test set). When judging performance in this way, using the *F*1 scores in isolation, the MARCO classifier outperforms CHiMP Classifier v1 on each class except ‘Other’ on both of the test sets (Supplementary Table S9). At the time of embarking on the work to replace the MARCO model and, in fact, immediately after training CHiMP Classifier v1, the model evaluation on the test sets had not been carried out and this performance difference was not known. In hindsight, the poorer performance of the MARCO model on the ‘Other’ class, coupled with the lower precision of the model on ‘Precipitate’ outcomes and a lower recall of ‘Crystal’ outcomes, may have led the VMXi beamline staff to prefer CHiMP Classifier v1 over the MARCO model despite the higher *F*1 scores for MARCO on these latter classes.

To enable comparison with other studies, the two classifier models were evaluated against the 47 029 validation images in the MARCO Data Set (full results are shown in Supplementary Tables S7 and S8). As described by Rosa *et al.* (2023 ▸), this data set may contain labelling errors, contamination from lipid cubic phase images and image redundancy. Given that our study focused on optimizing performance for local DLS data, our primary evaluation emphasizes the independent test sets from the VMXi beamline rather than this set. However, it is of note that CHiMP Classifier v2, unsurprisingly, performed better (*F*1 of 0.76 for ‘Crystals’) on this set of images when compared with CHiMP Classifier v1 (*F*1 of 0.48 for ‘Crystals’), as it was trained on both the MARCO and VMXi data sets. It is also of note that the low *F*1 scores for ‘Other’ highlight the potential discrepancy between how institutions define this ambiguous class. Conversely, the poor performance of CHiMP Classfier v1 on these data underscores the relatively strong performance of the MARCO classifier on VMXi images (Supplementary Table S9) despite none of these data having been used to train the model.

The improved performance of CHiMP Classifier v2 over CHiMP Classifier v1 on the ‘Crystals’ class was not apparent when solely observing statistics on the VMXi validation set (shown in Supplementary Tables S5 and S6); in fact, the precision, recall and *F*1 were all lower for the newer model. The superior performance of CHiMP Classifier v2 only becomes clear when measuring against the independent test sets. This again reinforces the importance of having a set of images categorized by experts to give a reliable measure of performance.

For the first version of the CHiMP classifier, the effectiveness of the ResNet50 model architecture and the training strategy used in this study are highlighted by the fact that the data set used to train the CHiMP Classifier v1 model comprised <2.7% of the number of images used to train the MARCO model. For the second version of the *CHiMP* classifier, a pretrained ConvNeXt-Tiny network was fine-tuned on the 415 775 training images from the MARCO Data Set before transfer learning was performed using the VMXi Classification Data Set, with the hypothesis that the extra training would yield an improvement over fine-tuning on VMXi data alone. This resulting improvement can be seen when comparing the ConvNeXt-V model (fine-tuned on VMXi data only) with CHiMP Classifier v2 in Supplementary Table S9; the model trained on less data achieved an average *F*1 metric of 0.58 on the unambiguous test set and 0.59 on the mostly unambiguous test set, whereas as CHiMP Classifier v2 achieved *F*1 metrics of 0.68 and 0.65, respectively, on the same test sets. When a ResNet50 model was fine-tuned with this additional data, in the same manner as the CHiMP Classifier v2 model, an improvement was also seen in the average *F*1 metric over the MARCO model, as well as for the ‘Crystals’ class, where the model achieved an *F*1 metric of 0.82 on the unambiguous test set and 0.71 on the mostly unambiguous test sets. This shows that it is not the ConvNeXt architecture alone that is able to outperform the MARCO model. After a ResNet50 model or a ConvNeXt-Tiny model has been trained on the MARCO Data Set alone, it achieves marginally higher average *F*1 metrics on the VMXi test sets than the MARCO classifier. Because the training strategies were different, including using weights from models pre-trained on ImageNet, this improvement cannot be put down to the model architecture alone. As a result of the strategy used here, the total number of training epochs required to train these models (nine epochs) is much lower than that used to train the MARCO classifier (260 epochs). This shows that using pre-trained weights has the advantage of achieving comparable model performance whilst using fewer computational resources and less energy.

### 5.2. On the detection of objects in experimental micrographs

Whereas the quality of label data from, sometimes ambiguous, experimental outcomes was often an issue when training classification networks, the quantity of label data was more of an issue when creating a tool to detect and segment instances of objects in the images. The effort involved in creating the inputs required to train a Mask R-CNN network, namely masks, labels and bounding boxes for every object in the images, were far greater than the single label per image input required for classification. As a result of this, a collaborative approach using the citizen-science platform Zooniverse was used (described in Section 3.2.2). Using this platform was a relatively straightforward way to create a web interface that allowed annotation of images by utilizing pre-existing drawing tools and allowed multiple annotators to work on the same data set simultaneously whilst maintaining data integrity. Even with the combined effort of several annotators, however, the size of the resulting data sets was small, with 237 images in the VMXi Detector Data Set and 350 images in the XChem Detector Data Set.

Much as for the classification networks, the limited amount of training data meant that a strategy of fine-tuning a set of pre-trained weights was used, along with the use of image augmentations to prevent overfitting to the data. The final mAP metrics (reported in Section 4.2.1 for the VMXi CHiMP Detector network and in Section 4.2.2 for the XChem CHiMP Detector network) show that the XChem CHiMP Detector achieved a higher mAP on both bounding boxes (0.38 versus 0.31) and segmentation (0.39 versus 0.29) for all objects when averaged over the standard IoU thresholds (0.5–0.95, with a step of 0.05). However, the mAP performance of the VMXi CHiMP Detector network was higher for large objects (>96 × 96 pixels) for both bounding boxes (0.66 versus 0.64) and segmentation (0.66 versus 0.61) over the same IoU thresholds than for the XChem Detector network. It is important to bear in mind that the mAP for the XChem Detector is averaged over two classes, ‘Crystals’ and ‘Drops’, whereas the VMXi Detector only predicts the ‘Crystals’ class.

The complexities of calculating mAP values mean that it is hard to make a direct comparison with the performance of the only other protein crystal detection network in the literature which also has associated metrics. Qin *et al.* (2021 ▸) reported a mAP score of 0.70 at an IoU threshold of 0.5 for their Mask R-CNN (presumably for bounding boxes, although this is not specified), which detects solely ‘Crystals’ class images from the MARCO Data Set. This is higher than the score that was achieved by the VMXi CHiMP Detector network using the same metric (0.57 for bounding boxes and 0.522 for segmentation). The figure reported in the study was calculated on just ten images and no breakdown of performance on different IoU thresholds and different object sizes is given, which further complicates a direct comparison.

The detector networks were evaluated on VMXi test data sets to calculate metrics comparable to those of the classifier networks (Tables 7 ▸ and 9 ▸). The *F*1 scores for classifying images into the ‘Crystals’ class (one or more crystals detected) are lower for these networks (0.58 for the VMXi CHiMP Detector, 0.62 for the XChem CHiMP Detector on the unambiguous set and 0.47 and 0.53, respectively, for the same networks on the mostly unambiguous set) than for the classifier networks (0.83 for CHiMP Classifier v2, 0.76 for CHiMP Classifier v1 and 0.78 for MARCO on the unambiguous set and 0.73, 0.66 and 0.69 for the same networks, respectively, on the mostly unambiguous set). However, the recall values are similar, or even better (for example, a ‘Crystals’ recall of 0.94 for the VMXi detector on the unambiguous set versus 0.90 for CHiMP Classifier v2), with low precision scores affecting *F*1 due to overdetection of crystals. It is worth noting that the VMXi CHiMP Detector was fine-tuned on just 180 images from the beamline, while the XChem CHiMP Detector was further trained on 281 images from the XChem facility, whereas the VMXi classification networks were fine-tuned on 11 161 images. Although these metrics provide insight, they are secondary since detection and classification networks serve different purposes. Detection involves labelling and segmenting potentially overlapping objects at various locations in the image. These candidate positions are subject to a thresholding selection, according to assigned confidence scores, and are also subject to overlap-suppression algorithms. Classification, meanwhile, focuses on recognizing the overall theme of an image without regard to object location or multiplicity. Different metrics, such as mAP, are more suitable for object detection. Future work might explore combining classifier and detector outputs for better crystal detection. Currently, both outputs are displayed alongside each other in the SynchWeb interface to allow scientists to view them simultaneously (Supplementary Fig. S3).

The utility of the VMXi CHiMP Detector network and the XChem CHiMP Detector network, in isolation, can be judged by viewing examples of their predicted output on images from their validation sets and comparing the output with the annotation provided by expert crystallographers. Figs. 5 ▸ and 6 ▸ show some of these examples for each of the detector networks. The predicted objects [panels (*b*), (*e*) and (*h*)] in these figures broadly agree with the expert annotation [panels (*c*), (*f*) and (*i*)]. There are some instances where crystals annotated by experts are missed (for example, Figs. 5 ▸*b*, 5 ▸*c*, 6 ▸*h* and 6 ▸*i*); however, there are also some instances where the network has annotated crystals that have been missed by the human (for example, Figs. 5 ▸*e* and 5 ▸*f* plus 5 ▸*h* and 5 ▸*i*). For the XChem CHiMP Detector, using the predicted drop and crystal masks to calculate a candidate position for dispensing compound appears to provide coordinates that are at a location away from the bulk of mountable crystals and also away from the edge of the drop.

## 6. Conclusions

The ultimate value of the automatic classification of images into categories of experimental outcome is realized when the model is integrated into the software interface that scientists use to monitor their experiments. In this case, a schematic overview of the subwells in the crystallization plate in the VMXi SynchWeb interface (Fig. 3 ▸) allows the locations of various outcomes to be quickly found along with a general overview of the proportion of experiments that resulted in each outcome. This overview of outcomes can then be used as the entry point to view images that are likely to be of interest and that have been further annotated by an object-detection network to pinpoint any crystals that are present. An example of this interface can be seen in Supplementary Fig. S3. The fact that the classification tool created in this study has been shown to achieve performance metrics that surpass those achieved by the MARCO tool (itself seen as one that could be trusted by crystallographers weary of missing important results) hopefully means that many hours otherwise spent performing manual inspection of images will be saved by this work. Integrating the VMXi CHiMP Detector tool into the SynchWeb interface has meant that human interaction is no longer required to mark the location of the crystals and add them to a queue for X-ray data collection. This in turn means that the experimental station at the beamline has the potential to work fully autonomously, with setting up the experiments in the crystal plate and adding the information to the ISPyB database being the only steps that require human intervention. After this has been performed, plate inspections, data collection and protein structure determination can now take place fully automatically.

By using the XChem CHiMP Detector network, candidate positions for the dispensing of small-molecule fragment solutions into the droplets can now be calculated automatically, thereby speeding up the process of setting up soaking experiments during screening campaigns, where hundreds of such compounds are soaked into crystals prior to X-ray data collection.

It is anticipated that sharing the training data, as well as the model weights and the code for model training and prediction of the networks described here, will aid other researchers to take advantage of these tools. The models can be used as shared, otherwise the model weights can be a starting point for transfer learning on local data. In order to make full use of these tools, work will, of course, need to be done to integrate the input images and model outputs into the existing software environment at the host institution.

Ultimately, it is hoped that this work will lead to faster structural discoveries that will have the potential to further our understanding of disease processes and how to treat them.

## 7. Related literature

The following references are cited in the supporting information for this article: Huang *et al.* (2016 ▸), Wang, Lee *et al.* (2022 ▸) and Wang, Sun *et al.* (2022 ▸).

## Supplementary Material

Initial installation and evaluation, Supplementary Methods and Supplementary Results, including Supplementary Figures and Tables. DOI: 10.1107/S2059798324009276/nj5327sup1.pdf

The 18 782 images from VMXi resulting from the initial database query alongside CSV files containing labels for the 13 951 images that make up the cleaned VMXi Classification Data Set.: https://doi.org/10.5281/zenodo.11097395

The images that make up the VMXi CHiMP Detector and XChem CHiMP Detector Data Sets, along with the corresponding image masks for drops and crystals.: https://doi.org/10.5281/zenodo.11110372

The model weights for the ConvNeXt-Tiny CHiMP Classifier v2 network.:  https://doi.org/10.5281/zenodo.11190973

The model weights for the Mask R-CNN VMXi CHiMP Detector network.: https://doi.org/10.5281/zenodo.11164787

The model weights for the Mask R-CNN XChem CHiMP Detector network.: https://doi.org/10.5281/zenodo.11165194

Software repository containing methods used for training and inference of the classification and detection networks.: https://doi.org/10.5281/zenodo.11244711

## Figures and Tables

**Table 1:** Mapping of VMXi image-scoring labels to a four-label system

Score	VMXi label	Mapped label
0	Clear	Clear
1	Contaminated	Other
2	Light Precipitate	Precipitate
3	Heavy Precipitate	Precipitate
4	Phase Separation	Other
5	Spherulites	Other
6	Microcrystals	Crystals
7	1D Crystals	Crystals
8	2D Crystals	Crystals
9	3D Crystals	Crystals

**Table 2:** Breakdown of image classes in the cleaned VMXi Classification Data Set

Label	Training set	Validation set	Total
Crystals	6752	1654	8406
Clear	1247	282	1529
Precipitate	2430	671	3101
Other	732	183	915
Total	11161	2790	13951

**Table 3:** Breakdown of image classes in the publicly released MARCO Data Set The data set is available from https://marco.ccr.buffalo.edu/download.

Label	Training set	Validation set	Total
Crystals	53044	6200	59244
Clear	139865	15659	155524
Precipitate	199567	22360	221927
Other	23299	2810	26109
Total	415775	47029	462804

**Table 4:** The number of images in each category for the VMXi classification test sets Sets were collated after the scoring of 1000 images by three experts. Unambiguous: images where three experts agreed on a label. Mostly unambiguous: images where two or more experts agreed on a label.

	Test set	
Label	Unambiguous	Mostly unambiguous
Crystals	145	179
Clear	95	182
Precipitate	230	356
Other	162	232
Total	632	949

**Table 5:** Per-class *F*1 metrics for test sets of images from the VMXi beamline Test set U denotes the unambiguous test set of 632 images where three experts agreed on the label. Test set MU denotes the mostly unambiguous test set of 949 images where two experts agreed on the label.

Model	Test set	Crystals	Precipitate	Clear	Other	Mean
*CHiMP* v2	U	0.8291	0.8221	0.6286	0.4286	0.6771
	MU	0.7340	0.7484	0.6823	0.4456	0.6526
*CHiMP* v1	U	0.7649	0.7411	0.6047	0.4710	0.6454
	MU	0.6635	0.6778	0.6415	0.4547	0.6094
MARCO	U	0.7759	0.7761	0.7179	0.1243	0.5986
	MU	0.6947	0.7346	0.7472	0.1473	0.5809

**Table 6:** Mean average precision metrics calculated for the VMXi CHiMP Detector Mask R-CNN for the ‘Crystals’ class Evaluated on the 47 validation images from the VMXi Detector Data Set.

Type	Threshold	Size (pixels)	mAP score
Bounding box	0.5	All	0.565
	0.75	All	0.307
	0.5–0.95, step 0.05	All	0.310
		<32 × 32	0.055
		32 × 32–96 × 96	0.428
		>96 × 96	0.657
Segmentation	0.5	All	0.522
	0.75	All	0.274
	0.5–0.95, step 0.05	All	0.288
		<32 × 32	0.026
		32 × 32–96 × 96	0.401
		>96 × 96	0.659

**Table 7:** Evaluation of the VMXi Detector network on the VMXi classification test sets The four image-classification categories were collapsed into ‘Crystals’ and ‘No crystals’ (‘Precipitate’, ‘Clear’ and ‘Other’). The model was deemed to classify the image as ‘Crystals’ if at least one crystal object was detected in the image. Test set U denotes the unambiguous test set of 632 images where three experts agreed on the label. Test set MU denotes the mostly unambiguous test set of 949 images where two experts agreed on the label.

Class	Test set	Precision	Recall	*F*1
Crystals	U	0.4177	0.9448	0.5793
	MU	0.3300	0.9218	0.4860
No crystals	U	0.9737	0.6078	0.7484
	MU	0.9688	0.5649	0.7137

**Table 8:** Mean average precision metrics calculated for the XChem CHiMP Detector Mask-R-CNN averaged for the ‘Crystals’ class and the ‘Drops’ class Evaluated on the 70 validation images in the XChem Detector Data Set.

Type	Threshold	Size (pixels)	mAP score
Bounding box	0.5	All	0.505
	0.75	All	0.381
	0.5–0.95, step 0.05	All	0.380
		<32 × 32	0.101
		32 × 32–96 × 96	0.214
		>96 × 96	0.641
Segmentation	0.5	All	0.467
	0.75	All	0.368
	0.5–0.95, step 0.05	All	0.386
		<32 × 32	0.081
		32 × 32–96 × 96	0.155
		>96 × 96	0.605

**Table 9:** Evaluation of the XChem Detector network on the VMXi classification test sets The four image-classification categories were collapsed into ‘Crystals’ and ‘No crystals’ (‘Precipitate’, ‘Clear’ and ‘Other’). The model was deemed to classify the image as ‘Crystals’ if at least one crystal object was detected in the image. Test set U denotes the unambiguous test set of 632 images where three experts agreed on the label. Test set MU denotes the mostly unambiguous test set of 949 images where two experts agreed on the label.

Class	Test set	Precision	Recall	*F*1
Crystals	U	0.4921	0.8551	0.6247
	MU	0.3945	0.8045	0.5294
No crystals	U	0.9447	0.7372	0.8281
	MU	0.9401	0.7130	0.8109

## Data Availability

We provide the set of 18 782 images from VMXi resulting from the initial database query (described in Section 3.1.1), alongside CSV files containing labels for the 13 951 images that make up the cleaned VMXi Classification Data Set. These can be found at https://doi.org/10.5281/zenodo.11097395 along with the 1000 VMXi images and the corresponding expert labels that form the test sets of images used to evaluate model performance. The images that make up the VMXi CHiMP Detector and XChem CHiMP Detector Data Sets, along with the corresponding image masks for drops and crystals, are available at https://doi.org/10.5281/zenodo.11110372. The model weights for the ConvNeXt-Tiny CHiMP Classifier v2 network are available at https://doi.org/10.5281/zenodo.11190973. The model weights for the Mask R-CNN VMXi CHiMP Detector network are available at https://doi.org/10.5281/zenodo.11164787. The model weights for the Mask R-CNN XChem CHiMP Detector network are available at https://doi.org/10.5281/zenodo.11165194. The software repository containing methods used for training and inference of the classification and detection networks discussed in this study is available at https://doi.org/10.5281/zenodo.11244711.

## References

[bb1] Beale, J. H., Bolton, R., Marshall, S. A., Beale, E. V., Carr, S. B., Ebrahim, A., Moreno-Chicano, T., Hough, M. A., Worrall, J. A. R., Tews, I. & Owen, R. L. (2019). *J. Appl. Cryst.* **52**, 1385–1396. 10.1107/S1600576719013517 PMC6878878 31798361

[bb2] Bischoff, D., Walla, B. & Weuster-Botz, D. (2022). *Anal. Bioanal. Chem.* **414**, 6379–6391. 10.1007/s00216-022-04101-8 PMC9372129 35661232

[bb3] Bradski, G. (2000). *Dr Dobb’s J. Softw. Tools*, **120**, 122–125.

[bb4] Bruno, A. E., Charbonneau, P., Newman, J., Snell, E. H., So, D. R., Vanhoucke, V., Watkins, C. J., Williams, S. & Wilson, J. (2018). *PLoS One*, **13**, e0198883. 10.1371/journal.pone.0198883 PMC6010233 29924841

[bb6] Cheng, Y. (2018). *Science*, **361**, 876–880. 10.1126/science.aat4346 PMC6460916 30166484

[bb7] Chollet, F. (2017). *2017 IEEE Conference on Computer Vision and Pattern Recognition (CVPR)*, pp. 1800–1807. Piscataway: IEEE.

[bb8] Cipriani, F., Felisaz, F., Launer, L., Aksoy, J.-S., Caserotto, H., Cusack, S., Dallery, M., di-Chiaro, F., Guijarro, M., Huet, J., Larsen, S., Lentini, M., McCarthy, J., McSweeney, S., Ravelli, R., Renier, M., Taffut, C., Thompson, A., Leonard, G. A. & Walsh, M. A. (2006). *Acta Cryst.* D**62**, 1251–1259. 10.1107/S0907444906030587 17001102

[bb9] Cumbaa, C. A. & Jurisica, I. (2010). *J. Struct. Funct. Genomics*, **11**, 61–69. 10.1007/s10969-009-9076-9 PMC2857471 20072819

[bb10] Cumbaa, C. A., Lauricella, A., Fehrman, N., Veatch, C., Collins, R., Luft, J. R., DeTitta, G. & Jurisica, I. (2003). *Acta Cryst.* D**59**, 1619–1627. 10.1107/s0907444903015130 12925793

[bb11] Delagenière, S., Brenchereau, P., Launer, L., Ashton, A. W., Leal, R., Veyrier, S., Gabadinho, J., Gordon, E. J., Jones, S. D., Levik, K. E., McSweeney, S. M., Monaco, S., Nanao, M., Spruce, D., Svensson, O., Walsh, M. A. & Leonard, G. A. (2011). *Bioinformatics*, **27**, 3186–3192. 10.1093/bioinformatics/btr535 21949273

[bb12] Douangamath, A., Powell, A., Fearon, D., Collins, P. M., Talon, R., Krojer, T., Skyner, R., Brandao-Neto, J., Dunnett, L., Dias, A., Aimon, A., Pearce, N. M., Wild, C., Gorrie-Stone, T. & von Delft, F. (2021). *J. Vis. Exp.*, e62414. 10.3791/62414 34125095

[bb76] Edwards, D. W. II & Dinc, I. (2020). *CSBio’20: Proceedings of the Eleventh International Conference on Computational Systems – Biology and Bioinformatics*, pp. 54–60. New York: Association for Computing Machinery.

[bb13] Fischer, M. (2021). *Q. Rev. Biophys.* **54**, e1. 10.1017/S0033583520000128 33413726

[bb14] Fisher, S. J., Levik, K. E., Williams, M. A., Ashton, A. W. & McAuley, K. E. (2015). *J. Appl. Cryst.* **48**, 927–932. 10.1107/S1600576715004847 PMC4453979 26089766

[bb15] Formulatrix (2019). *Protein Crystallization Software Update: ROCK MAKER 3.15*. https://formulatrix.com/life-science-automation-blog/protein-crystallization-software-update-rock-maker-3-15/.

[bb16] Gao, Z., Wu, Y., Bao, Y., Gong, J., Wang, J. & Rohani, S. (2018). *Cryst. Growth Des.* **18**, 4275–4281.

[bb17] Ghafurian, S., Orth, P., Strickland, C., Su, H., Patel, S., Soisson, S. & Dogdas, B. (2018). *arXiv*:1805.04563.

[bb18] Gildea, R. J., Beilsten-Edmands, J., Axford, D., Horrell, S., Aller, P., Sandy, J., Sanchez-Weatherby, J., Owen, C. D., Lukacik, P., Strain-Damerell, C., Owen, R. L., Walsh, M. A. & Winter, G. (2022). *Acta Cryst.* D**78**, 752–769. 10.1107/S2059798322004399 PMC9159281 35647922

[bb19] Han, J., Ding, J., Li, J. & Xia, G.-S. (2022). *IEEE Trans. Geosci. Remote Sensing*, **60**, 5602511.

[bb20] He, K., Gkioxari, G., Dollár, P. & Girshick, R. (2020). *IEEE Trans. Pattern Anal. Mach. Intell.* **42**, 386–397. 10.1109/TPAMI.2018.2844175 29994331

[bb21] He, K., Zhang, X., Ren, S. & Sun, J. (2015). *2015 IEEE International Conference on Computer Vision (ICCV)*, pp. 1026–1034. Piscataway: IEEE.

[bb22] He, K., Zhang, X., Ren, S. & Sun, J. (2016). *2016 IEEE Conference on Computer Vision and Pattern Recognition (CVPR)*, pp. 770–778. Piscataway: IEEE.

[bb23] Healey, R. D., Basu, S., Humm, A.-S., Leyrat, C., Cong, X., Golebiowski, J., Dupeux, F., Pica, A., Granier, S. & Márquez, J. A. (2021). *Cell Rep. Methods*, **1**, 100102. 10.1016/j.crmeth.2021.100102 PMC8545655 34723237

[bb24] Hough, P. V. C. (1962). US Patent US3069654A.

[bb25] Howard, J. & Gugger, S. (2020). *Information*, **11**, 108.

[bb26] Huang, G., Liu, Z., Maaten, L. V. D. & Weinberger, K. Q. (2017). *2017 IEEE Conference on Computer Vision and Pattern Recognition (CVPR)*, pp. 2261–2269. Piscataway: IEEE.

[bb27] Huang, G., Sun, Y., Liu, Z., Sedra, D. & Weinberger, K. Q. (2016). *Computer Vision – ECCV 2016*, edited by B. Leibe, J. Matas, N. Sebe & M. Welling, Part IV, pp. 646–661. Cham: Springer.

[bb28] Iandola, F. N., Han, S., Moskewicz, M. W., Ashraf, K., Dally, W. J. & Keutzer, K. (2016). *arXiv*:1602.07360.

[bb29] Ito, S., Ueno, G. & Yamamoto, M. (2019). *J. Synchrotron Rad.* **26**, 1361–1366. 10.1107/S160057751900434X PMC6613109 31274465

[bb30] Jancarik, J. & Kim, S.-H. (1991). *J. Appl. Cryst.* **24**, 409–411.

[bb31] Jumper, J., Evans, R., Pritzel, A., Green, T., Figurnov, M., Ronneberger, O., Tunyasuvunakool, K., Bates, R., Žídek, A., Potapenko, A., Bridgland, A., Meyer, C., Kohl, S. A. A., Ballard, A. J., Cowie, A., Romera-Paredes, B., Nikolov, S., Jain, R., Adler, J., Back, T., Petersen, S., Reiman, D., Clancy, E., Zielinski, M., Steinegger, M., Pacholska, M., Berghammer, T., Bodenstein, S., Silver, D., Vinyals, O., Senior, A. W., Kavukcuoglu, K., Kohli, P. & Hassabis, D. (2021). *Nature*, **596**, 583–589. 10.1038/s41586-021-03819-2 PMC8371605 34265844

[bb32] Kohonen, T. (1982). *Biol. Cybern.* **43**, 59–69.

[bb33] Krizhevsky, A., Sutskever, I. & Hinton, G. E. (2012). *Advances in Neural Information Processing Systems 25 (NIPS 2012)*, edited by F. Pereira, C. J. Burges, L. Bottou & K. Q. Weinberger, pp. 1097–1105. Red Hook: Curran Associates.

[bb34] Larsen, P. A., Rawlings, J. B. & Ferrier, N. J. (2006). *Chem. Eng. Sci.* **61**, 5236–5248.

[bb35] Lazo, E. O., Antonelli, S., Aishima, J., Bernstein, H. J., Bhogadi, D., Fuchs, M. R., Guichard, N., McSweeney, S., Myers, S., Qian, K., Schneider, D., Shea-McCarthy, G., Skinner, J., Sweet, R., Yang, L. & Jakoncic, J. (2022). *J. Synchrotron Rad.* **29**, 280. 10.1107/S1600577521013205 PMC8733988 34985446

[bb36] Lin, T.-Y., Maire, M., Belongie, S., Hays, J., Perona, P., Ramanan, D., Dollár, P. & Zitnick, C. L. (2014). *Computer Vision – ECCV 2014*, edited by D. Fleet, T. Pajdla, B. Schiele & T. Tuytelaars, pp. 740–755. Cham: Springer.

[bb37] Liu, R., Freund, Y. & Spraggon, G. (2008). *Acta Cryst.* D**64**, 1187–1195. 10.1107/S090744490802982X PMC2585161 19018095

[bb38] Liu, Z., Mao, H., Wu, C.-Y., Feichtenhofer, C., Darrell, T. & Xie, S. (2022). *2022 IEEE/CVF Conference on Computer Vision and Pattern Recognition (CVPR)*, pp. 11966–11976. Piscataway: IEEE.

[bb39] Loshchilov, I. & Hutter, F. (2017).* arXiv*:1711.05101.

[bb40] McAuley, K. E., Williams, M. & Fisher, S. (2015). *BART – The New Robotic Sample Changer for MX Beamlines at Diamond*. https://www.diamond.ac.uk/Home/Corporate-Literature/Annual-Review/Review2015/Villages/Macromolecular-Crystallography-Village/Macromolecular-Crystallography-Village-Developments/BART---the-new-robotic-sample-changer-for-MX-beamlines-at-Diamond.html.

[bb41] Milne, J., Qian, C., Hargreaves, D., Wang, Y. & Wilson, J. (2023). *PLoS One*, **18**, e0282562. 10.1371/journal.pone.0282562 PMC9997964 36893084

[bb42] Miura, Y., Sakurai, T., Aranha, C., Senda, T., Kato, R. & Yamada, Y. (2018). *arXiv*:1812.10087.

[bb43] Moreno-Chicano, T., Carey, L. M., Axford, D., Beale, J. H., Doak, R. B., Duyvesteyn, H. M. E., Ebrahim, A., Henning, R. W., Monteiro, D. C. F., Myles, D. A., Owada, S., Sherrell, D. A., Straw, M. L., Šrajer, V., Sugimoto, H., Tono, K., Tosha, T., Tews, I., Trebbin, M., Strange, R. W., Weiss, K. L., Worrall, J. A. R., Meilleur, F., Owen, R. L., Ghiladi, R. A. & Hough, M. A. (2022). *IUCrJ*, **9**, 610–624. 10.1107/S2052252522006418 PMC9438502 36071813

[bb44] Ng, J. T., Dekker, C., Kroemer, M., Osborne, M. & von Delft, F. (2014). *Acta Cryst.* D**70**, 2702–2718. 10.1107/S1399004714017581 PMC4188010 25286854

[bb45] Ng, J. T., Dekker, C., Reardon, P. & von Delft, F. (2016). *Acta Cryst.* D**72**, 224–235. 10.1107/S2059798315024687 PMC4756611 26894670

[bb46] Paszke, A., Gross, S., Massa, F., Lerer, A., Bradbury, J., Chanan, G., Killeen, T., Lin, Z., Gimelshein, N., Antiga, L., Desmaison, A., Kopf, A., Yang, E., DeVito, Z., Raison, M., Tejani, A., Chilamkurthy, S., Steiner, B., Fang, L., Bai, J. & Chintala, S. (2019). *Advances in Neural Information Processing Systems 32*, edited by H. Wallach, H. Larochelle, A. Beygelzimer, F. d’Alché-Buc, E. Fox & R. Garnett, pp. 8024–8035. Red Hook: Curran Associates.

[bb47] Pizer, S. M., Amburn, E. P., Austin, J. D., Cromartie, R., Geselowitz, A., Greer, T., ter Haar Romeny, B., Zimmerman, J. B. & Zuiderveld, K. (1987). *Comput. Vis. Graph. Image Process.* **39**, 355–368.

[bb48] Pohl, E., Ristau, U., Gehrmann, T., Jahn, D., Robrahn, B., Malthan, D., Dobler, H. & Hermes, C. (2004). *J. Synchrotron Rad.* **11**, 372–377. 10.1107/S090904950401516X 15310952

[bb49] Pons, M.-N. & Vivier, H. (1990). *Anal. Chim. Acta*, **238**, 243–249.

[bb50] Qin, J., Zhang, Y., Zhou, H., Yu, F., Sun, B. & Wang, Q. (2021). *Crystals*, **11**, 157.

[bb51] Ren, Z., Wang, C., Shin, H., Bandara, S., Kumarapperuma, I., Ren, M. Y., Kang, W. & Yang, X. (2020). *IUCrJ*, **7**, 1009–1018. 10.1107/S2052252520011288 PMC7642789 33209315

[bb52] Ronneberger, O., Fischer, P. & Brox, T. (2015). *Medical Image Computing and Computer-Assisted Intervention – MICCAI 2015*, edited by N. Navab, J. Hornegger, W. M. Wells & A. F. Frangi, pp. 234–241. Cham: Springer.

[bb53] Rosa, N., Watkins, C. J. & Newman, J. (2023). *PLoS One*, **18**, e0283124. 10.1371/journal.pone.0283124 PMC10038243 36961775

[bb54] Russakovsky, O., Deng, J., Su, H., Krause, J., Satheesh, S., Ma, S., Huang, Z., Karpathy, A., Khosla, A., Bernstein, M., Berg, A. C. & Fei-Fei, L. (2015). *Int. J. Comput. Vis.* **115**, 211–252.

[bb55] Sanchez-Weatherby, J., Sandy, J., Mikolajek, H., Lobley, C. M. C., Mazzorana, M., Kelly, J., Preece, G., Littlewood, R. & Sørensen, T. L.-M. (2019). *J. Synchrotron Rad.* **26**, 291–301. 10.1107/S1600577518015114 PMC6337891 30655497

[bb56] Schurmann, J., Lindhè, I., Janneck, J. W., Lima, G. & Matej, Z. (2019). *2019 53rd Asilomar Conference on Signals, Systems, and Computers*, pp. 978–983. Piscataway: IEEE.

[bb57] Selvaraju, R. R., Cogswell, M., Das, A., Vedantam, R., Parikh, D. & Batra, D. (2017). *2017 IEEE International Conference on Computer Vision (ICCV)*, pp. 618–626. Piscataway: IEEE.

[bb58] Simonyan, K. & Zisserman, A. (2014). *arXiv*:1409.1556.

[bb59] Simpson, R., Page, K. R. & De Roure, D. (2014). *Proceedings of the 23rd International Conference on World Wide Web*, pp. 1049–1054. New York: Association for Computing Machinery.

[bb60] Smith, L. N. & Topin, N. (2019). *Proc. SPIE*, **11006**, 1100612.

[bb61] Snell, E. H., Luft, J. R., Potter, S. A., Lauricella, A. M., Gulde, S. M., Malkowski, M. G., Koszelak-Rosenblum, M., Said, M. I., Smith, J. L., Veatch, C. K., Collins, R. J., Franks, G., Thayer, M., Cumbaa, C., Jurisica, I. & DeTitta, G. T. (2008). *Acta Cryst.* D**64**, 1123–1130. 10.1107/S0907444908028047 PMC2631114 19020350

[bb62] Song, J., Mathew, D., Jacob, S. A., Corbett, L., Moorhead, P. & Soltis, S. M. (2007). *J. Synchrotron Rad.* **14**, 191–195. 10.1107/S0909049507004803 17317920

[bb63] Spraggon, G., Lesley, S. A., Kreusch, A. & Priestle, J. P. (2002). *Acta Cryst.* D**58**, 1915–1923. 10.1107/s0907444902016840 12393922

[bb64] Strutz, T. (2021). *arXiv*:2106.03503.

[bb65] Szegedy, C., Vanhoucke, V., Ioffe, S., Shlens, J. & Wojna, Z. (2016). *2016 IEEE Conference on Computer Vision and Pattern Recognition (CVPR)*, pp. 2818–2826. Piscataway: IEEE.

[bb66] Tan, M. & Le, Q. V. (2019). *arXiv*:1905.11946.

[bb67] Thielmann, Y., Luft, T., Zint, N. & Koepke, J. (2023). *Acta Cryst.* A**79**, 331–338. 10.1107/S2053273323001948 PMC10317135 37265048

[bb68] Thorne, R. E. (2023). *Acta Cryst.* D**79**, 78–94. 10.1107/S2059798322011652 PMC9815097 36601809

[bb69] Wada, K. (2023). *Labelme: Image Polygonal Annotation with Python*. https://github.com/wkentaro/labelme.

[bb70] Wang, K., Lee, S., Balewski, J., Sim, A., Nugent, P., Agrawal, A., Choudhary, A., Wu, K. & Liao, W. K. (2022). *2022 22nd IEEE International Symposium on Cluster, Cloud and Internet Computing (CCGrid)*, pp. 404–413. Piscataway: IEEE.

[bb71] Wang, Y., Sun, D., Chen, K., Lai, F. & Chowdhury, M. (2022). *arXiv*:2201.06227.

[bb72] Ward, K. B., Perozzo, M. A. & Zuk, W. M. (1988). *J. Cryst. Growth*, **90**, 325–339.

[bb73] Wasserman, S. R., Benach, J., Koss, J. W. & Morisco, L. L. (2015). *Synchrotron Radiat. News* **28**(6), 4–9.

[bb74] Watts, D., Cowtan, K. & Wilson, J. (2008). *J. Appl. Cryst.* **41**, 8–17.

[bb75] Wightman, R. (2019). *PyTorch Image Models*. https://github.com/rwightman/pytorch-image-models.

[bb77] Yann, M. & Tang, Y. (2016). *Proc. AAAI Conf. Artif. Intell.* **30**, https://doi.org/10.1609/aaai.v30i1.10150.

[bb78] Zuk, W. M. & Ward, K. B. (1991). *J. Cryst. Growth*, **110**, 148–155.

