# Supplementary material for: CHiMP: deep-learning tools trained on protein crystallization micrographs to enable automation of experiments
Source: Acta Crystallogr D Struct Biol. 2024 Oct 1;80(Pt 10):744–64. doi: 10.1107/S2059798324009276 (PMC11448919; doi:10.1107/S2059798324009276)
Supplement: Supplementary file 1 [file d-80-00744-sup1.pdf]

STRUCTURAL
BIOLOGY

Volume 80 (2024)

Supporting information for article:

CHiMP: deep-learning tools trained on protein crystallization micrographs to enable automation of experiments

Oliver N. F. King, Karl E. Levik, James Sandy and Mark Basham

S1. Initial Installation and Evaluation of MARCO at the DLS VMXi Beamline

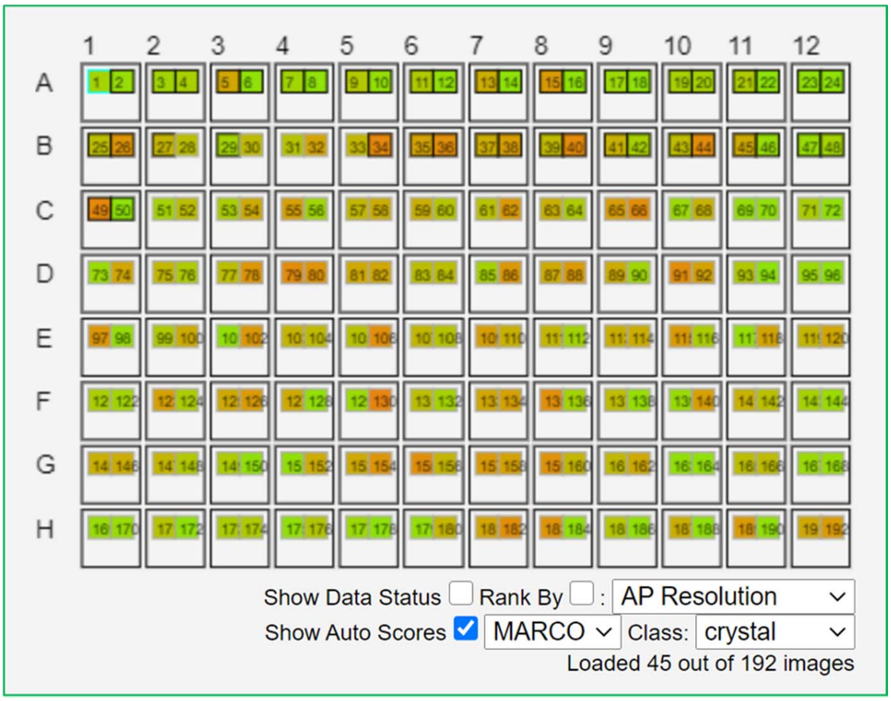

Figure S1 Schematic overview of classification outputs from the MARCO CNN (Bruno *et al.*, 2018), as displayed in SynchWeb (Fisher *et al.*, 2015) for users of the VMXi beamline at Diamond Light Source. The 96 plate wells are represented as larger boxes with the two subwells containing crystallisation droplets shown as smaller boxes within. Each subwell is assigned a colour on a gradient from red to green that represents the probability of the associated image being assigned to the class *Crystals* with green representing a high probability.

S1.1. Initial assessment of MARCO performance on images from the VMXi facility

To evaluate the performance of the MARCO model on micrographs captured in-house, the images from two crystal plates, each with 288 subwells was scored by an expert using the same four categories used by MARCO: *Crystals*, *Precipitate*, *Clear* and *Other*. One plate was from an experiment set-up to crystallise haemoglobin and the other contained thermolysin. From the 576 classified images, 20 were selected at random from each of the four classes giving a final, balanced, dataset of 80 images. These were then scored using MARCO for comparison, giving a precision, recall and $F1$ score for the *Crystals* class of 0.82, 0.7 and 0.76 respectively. The recall and $F1$ metric scores are moderately lower than those found by (Bruno *et al.*, 2018) for their independent test set of images (precision 0.78, a recall of 0.87 and $F1$ score of 0.82) but the precision is comparable. It was found that MARCO also tended to over-classify images as Precipitate, with 22 false positive classifications. This resulted in a recall of 100% of the *Precipitate* images but this was at the expense of precision which was 0.48 ($F1$ metric 0.65). Full metrics can be seen in Table S1.

Table S1 Per-class classification performance of the MARCO Classifier on a limited dataset of 80 images from two crystallisation plates, with 20 images selected at random from each class.

Class	Precision	Recall	F1
**Crystals**	0.8235	0.7000	0.7568
**Clear**	0.8000	0.6000	0.6857
**Precipitate**	0.4762	1.0000	0.6452
**Other**	1.0000	0.3000	0.4615

S2. Supplementary Methods

S2.1. Details on the collection of images at the VMXi beamline experimental facility

All experimental crystal plates on the VMXi beamline are registered as *containers* in the ISPyB LIMS relational database before being inserted into a Rock Imager 1000 (Formulatrix, USA) automated microplate imager. Images of every crystallisation sub-well are recorded (termed an *inspection*) immediately after insertion of the experimental plate and, in addition, subsequent inspections are carried out on a Fibonacci sequence schedule (i.e. 1, 3, 5, 8 ... days) to monitor the experiments over time. A z-stack of images, taken with different focal points within the plate subwell, is combined into an extended focus image and then saved as JPEG format with a resolution of 3376 × 2704 pixels. Once created, all images are moved to directories linked to the experimental visit that the container is part of and also to the inspection number. This information is also recorded in the ISPyB LIMS (Delagenière *et al.*, 2011). Once recorded, the images are available to view in the SynchWeb browser interface (Fisher *et al.*, 2015). In this interface, scientists are able to browse the images and provide a score for classifying the content of the image.

S2.2. Cleaning of the data labels for the VMXi Classification Dataset

A ResNet50 model initialised with ImageNet weights was fine-tuned with a random split of 80% of images in the training set and 20% of images in the validation set. The *ImageCleaner* functionality of the *fastai* library (Howard & Gugger, 2020) was then used to correct mislabelled images. This process entails using the trained ResNet50 to classify the entire dataset of images (training and validation set) whilst also recording the cross-entropy loss for the classifications. Since a high loss value for a classification can be equated with a high uncertainty, images can then be viewed in order of the most uncertain label first. The original label given in the dataset is displayed alongside the image and the label can then be corrected if it is wrong; this is more time-efficient than reviewing the entire dataset.

Table S2 Summary of label changes during cleaning of the VMXi Classification Dataset.

Number Changed is the number of images in that class that have a different label after cleaning to as opposed to before cleaning (rather than the difference in count for the entire category, as given in the Before Cleaning and After Cleaning columns). "Percentage Changed|" is calculated as a percentage of the label count before cleaning.

Label	Before Cleaning	After Cleaning	Number Changed	Percentage Changed
**Crystals**	8,301	8,406	249	3.0%
**Clear**	1,514	1,529	146	9.6%
**Precipitate**	3,037	3,101	338	11.1%
**Other**	1,099	915	171	15.6%
**Total**	**13,951**	**13,951**	**904**	**6.5%**

S2.3. Training the CHiMP Classifier-V1 ResNet50 CNN

At the start of each phase of model training, the weights of the ResNet50 convolutional layers were "frozen", i.e. gradients were not calculated, and the weights were not updated, only the weights of the fully connected classification head network, responsible for determining the posterior probability of the four image classes were available to be modified. Updating solely these parameters to allow learning of the specific output classes of the new task is faster than fine-tuning the entire model and preserves the pre-trained ImageNet weights in the other layers of the model. In addition, early adjustment of those features that are still relevant is avoided, which serves to help prevent the model from converging too quickly to a poor local minimum. More complex methods exist for selectively freezing the front layers of the network once they have converged in order to speed up training (Wang, Sun*, et al.*, 2022), but use of these such strategies was deemed unnecessary for the relatively small-scale fine-tuning task carried out here. After five epochs of fine-tuning on the partially frozen model, all the model weights were "unfrozen" allowing them to be updated during the fine-tuning process.

The learning rate to be used was determined through a process of training the model for one epoch whilst exponentially increasing the learning rate for each batch and plotting a graph of loss against learning rate. The rate is chosen from this graph according to the loss profile and this value is then used to define a range of values to be used in a "1Cycle" training strategy (Smith & Topin, 2019). In this strategy, which has been shown to accelerate model convergence, the learning rate is gradually increased to a maximum value during the middle of the training epochs and reduced to a low value

towards the end. Model parameter optimisation was carried out using AdamW (Loshchilov & Hutter, 2017).

In the first phase of training, an image size of 128 × 128 pixels was used followed by 256 × 256 pixels in the second phase and 512 × 512 pixels for the final phase. Training on smaller images is less computationally expensive and therefore faster. This speed up, coupled with the ability of the network to transfer learning from the coarser data through to the model trained on higher resolution data leads to an overall saving in terms of time to convergence (Wang, Lee*, et al.*, 2022). Using low resolution data in the early stages can also have the advantage of preventing the model from overfitting to small, irrelevant details and to ensure that the model parameters are guided toward reasonable initial values before being optimised on higher resolution information. This strategy is summarised in Table S3.

Table S3 Summary of the training phases for the CHiMP Classifier-v1 ResNet50 CNN using the VMXi Classification Dataset.

Image dimensions in pixels, F/U refers to Frozen or Unfrozen model weights respectively. Av Learn Rate refers to average learning rate used during a cyclic learning rate strategy. Loss is cross entropy loss.

Phase	Image Dim	Stage	F/U	Epochs	Av. Learn Rate	Train Loss	Valid Loss
**1**	128 × 128	1	F	5	5.2×10^{-3}	0.5743	0.6594
		2	U	5	5.25×10^{-5}	0.3675	0.5734
**2**	256 × 256	1	F	5	2.6×10^{-3}	0.3174	0.6648
		2	U	5	5.25×10^{-5}	0.1846	0.4670
**3**	512 × 512	1	F	5	1.3×10^{-3}	0.2301	0.4246
		2	U	6	5.25×10^{-5}	0.1147	0.3635

S2.4. Training the CHiMP Classifier-V2 ConvNeXt Tiny CNN

Initial fine-tuning of the model on the MARCO Dataset was done for 12 epochs in total, split across three phases with increasing image dimensions at each phase (starting with 128 × 128 pixels, then 256 × 256 pixels, then 512 × 512 pixels). Training and validation sets of data were the same as those publicly released by (Bruno *et al.*, 2018) and used to train their MARCO classification model (a breakdown of image classes can be seen in Table 3). The batch size used was 24. Due to the relatively large size of this dataset, training was distributed across four NVIDA Tesla V100S GPUs, each equipped with 32 GB of VRAM. The cross-entropy loss for the training set was 0.201 and for the validation set was 0.214 after this section of training process.

This model was then fine-tuned further on the VMXi Classification dataset (described in Section 3.1.1). During this process, the model was found to have a propensity to over-fit on this smaller dataset, with the training loss dropping much lower than the validation loss. As a result, more regularisation was added in the form of stochastic depth (Huang *et al.*, 2016) with a probability of 0.2. In this technique, layers of the network are randomly dropped during training, shortening the network and forcing the model to adapt to a situation where not all layers are used, this allows the model to be trained for longer without over-fitting. A two-phase strategy was used with first phase using images with dimensions of 256 × 256 pixels for 10 epochs. The model weights were frozen for the first epoch, training only the network head. In the second phase, using images of 512 × 512 pixels, the model was fine-tuned for 14 epochs. The final training loss was 0.421 and the final validation loss was 0.522.

Table S4 Summary of the training phases for the CHiMP Classifier-v2 ConvNeXt Tiny CNN using the MARCO dataset (M) and then the VMXi Classification Dataset (V).

Image dimensions in pixels, F/U refers to Frozen or Unfrozen model weights respectively. Av Learn Rate refers to average learning rate used during a cyclic learning rate strategy. Loss is cross entropy loss.

Phase	Image Dim	Data	F/U	Epochs	Av. Learn Rate	Train Loss	Valid Loss
**1**	128 × 128	M	F	1	3.9×10^{-4}	0.9586	0.7380
		M	U	3	2.6×10^{-5}	0.3722	0.3026
**2**	256 × 256	M	U	4	2.6×10^{-5}	0.1939	0.2422
**3**	512 × 512	M	U	4	2.6×10^{-5}	0.2013	0.2136
**4**	256 × 256	V	F	1	2.6×10^{-5}	1.5590	1.7364
		V	U	9	2.6×10^{-5}	0.6810	0.6634
**5**	512 × 512	V	U	14	2.6×10^{-5}	0.4206	0.5220

S2.4.1. Creating image annotations for detection networks

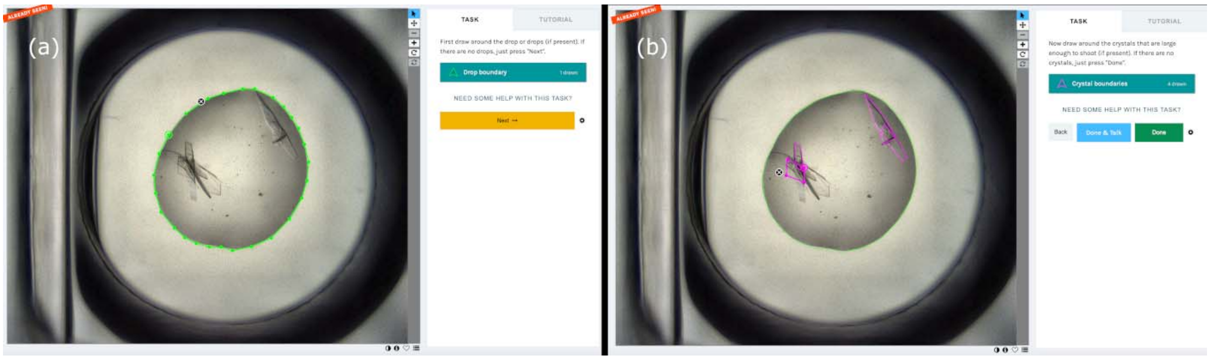

Figure S2 The Zooniverse annotation interface for generating training data for networks that perform object detection and instance segmentation. (a) An example of annotating a crystallisation drop. (b) An example of annotating crystals.

S3. Supplementary Results

S3.1. Performance of networks on the VMXi Classification Dataset validation set

Table S5 and Table S6 show the per-class metrics for CHiMP Classifier-v1 and CHiMP Classifier-v2 respectively on the validation images from the VMXi Classification Dataset.

Table S5 Per-class classification performance of CHiMP Classifier-v1 on validation images from the VMXi Classification Dataset

Class	Precision	Recall	F1
**Crystals**	0.9525	0.9208	0.9367
**Clear**	0.8392	0.9255	0.8803
**Precipitate**	0.8431	0.8569	0.8500
**Other**	0.5856	0.6339	0.6089

Table S6 Per-class classification performance of CHiMP Classifier-v2 on validation images from the VMXi Classification Dataset

Class	Precision	Recall	F1
**Crystals**	0.9701	0.8839	0.9250
**Clear**	0.8457	0.9326	0.8870
**Precipitate**	0.8560	0.8331	0.8444
**Other**	0.5297	0.9235	0.6733

S3.2. Performance of networks on the MARCO Dataset validation set

Table S7 and Table S8 show the per-class metrics for CHiMP Classifier-v1 and CHiMP Classifier-v2 respectively on the 47,029 validation images from the MARCO Dataset.

Table S7 Per-class classification performance of CHiMP Classifier-v1 on validation images from the MARCO Dataset

Class	Precision	Recall	F1
**Crystals**	0.3847	0.6226	0.4755
**Clear**	0.6920	0.5457	0.6102
**Precipitate**	0.6916	0.6528	0.6716
**Other**	0.2217	0.2794	0.2472

Table S8 Per-class classification performance of CHiMP Classifier-v2 on validation images from the MARCO Dataset

Class	Precision	Recall	F1
**Crystals**	0.7070	0.8282	0.7628
**Clear**	0.9109	0.7974	0.8503
**Precipitate**	0.9514	0.8238	0.8830
**Other**	0.3317	0.7907	0.4674

S3.3. Classification Performance on Test Sets of Images from the VMXi Beamine

Performance metrics for several different classification models on the test sets of images (described in Section 3.1.5) are shown here.

Table S9 Per-class precision, recall and *F*1 metrics for classification models for test sets of images from the VMXi beamline.

Test Set U denotes the unambiguous test set of 632 images where three experts agreed on the label. Test Set MU denotes the mostly unambiguous test set of 949 images where two experts agreed on the label. Models **CHiMP Classifier-v1**, **CHiMP Classifier-v2** and **MARCO** as described in main text. **ResNet50-MV** model trained on both MARCO and VMXi classification datasets in the same fashion as CHiMP-v2. **ResNet50-M** model trained solely on MARCO Classification Dataset (prior to additional training on the VMXi Classification Dataset to create ResNet50-MV). **ConvNeXt-M** model trained solely on MARCO Classification Dataset (prior to additional training on the VMXi Classification Dataset to create CHiMP Classifier-v2). **ConvNeXt-V** model trained solely on the VMXi Classification Dataset.

		**Crystals**			**Precipitate**			**Clear**			**Other**			**Average**		
**Model**	**T-Set**	**Prec**	**Rec**	**F1**	**Prec**	**Rec**	**F1**	**Prec**	**Rec**	**F1**	**Prec**	**Rec**	**F1**	**Prec**	**Rec**	**F1**
**CHiMP-v2**	U	0.7661	0.9034	0.8291	0.9194	0.7435	0.8221	0.4757	0.9263	0.6286	0.6000	0.3333	0.4286	**0.6903**	**0.7266**	**0.6771**
	MU	0.6564	0.8324	0.7340	0.8640	0.6601	0.7484	0.5575	0.8791	0.6823	0.5399	0.3793	0.4456	**0.6544**	**0.6877**	**0.6526**
**ResNet50-MV**	U	0.7651	0.8759	0.8167	0.8557	0.7478	0.7981	0.4886	0.9053	0.6347	0.5393	0.2963	0.3825	**0.6622**	**0.7063**	**0.6580**
	MU	0.6495	0.7765	0.7074	0.8182	0.6573	0.7290	0.5477	0.8516	0.6667	0.4759	0.3405	0.3970	**0.6228**	**0.6565**	**0.6250**
**CHiMP-v1**	U	0.7011	0.8414	0.7649	0.8902	0.6348	0.7411	0.4785	0.8211	0.6047	0.5267	0.4259	0.4710	**0.6492**	**0.6808**	**0.6454**
	MU	0.5761	0.7821	0.6635	0.8354	0.5702	0.6778	0.5620	0.7473	0.6415	0.4661	0.4440	0.4547	**0.6099**	**0.6359**	**0.6094**
**ResNet50-M**	U	0.6000	0.7862	0.6806	0.8316	0.6870	0.7524	0.5111	0.9684	0.6691	0.5972	0.2654	0.3675	**0.6350**	**0.6768**	**0.6174**
	MU	0.5316	0.7039	0.6058	0.7860	0.5983	0.6794	0.5475	0.9505	0.6948	0.5440	0.2931	0.3810	**0.6023**	**0.6365**	**0.5902**
**ConvNeXt-M**	U	0.5043	0.8069	0.6207	0.8660	0.7304	0.7925	0.5506	0.9158	0.6877	0.6875	0.2037	0.3143	**0.6521**	**0.6642**	**0.6038**
	MU	0.4596	0.7318	0.5647	0.7930	0.6348	0.7051	0.5700	0.9176	0.7032	0.6163	0.2284	0.3333	**0.6097**	**0.6282**	**0.5766**
**MARCO**	U	0.7532	0.8000	0.7759	0.6636	0.9348	0.7762	0.6043	0.8842	0.7179	0.7333	0.0679	0.1243	**0.6886**	**0.6717**	**0.5986**
	MU	0.6966	0.6927	0.6947	0.6352	0.8708	0.7346	0.6381	0.9011	0.7472	0.7308	0.0819	0.1473	**0.6752**	**0.6366**	**0.5809**
**ConvNeXt-V**	U	0.5789	0.8345	0.6836	0.8667	0.5652	0.6842	0.4940	0.8737	0.6312	0.4381	0.2840	0.3446	**0.5944**	**0.6393**	**0.5859**
	MU	0.5000	0.7765	0.6083	0.7932	0.5281	0.6341	0.5659	0.8022	0.6636	0.4148	0.3147	0.3578	**0.5685**	**0.6054**	**0.5660**

S3.4. SynchWeb visualisation of the output of the classification and detection networks for users of the VMXi experimental facility

The outputs from the CHiMP Classifier-v2 network and the VMXi CHiMP detector network are stored in the ISPyB LIMS. Using the SynchWeb web interface, facility users can browse this information in the form of a plate overview schematic alongside images with overlaid crystal centroids that are displayed by clicking on a subwell.

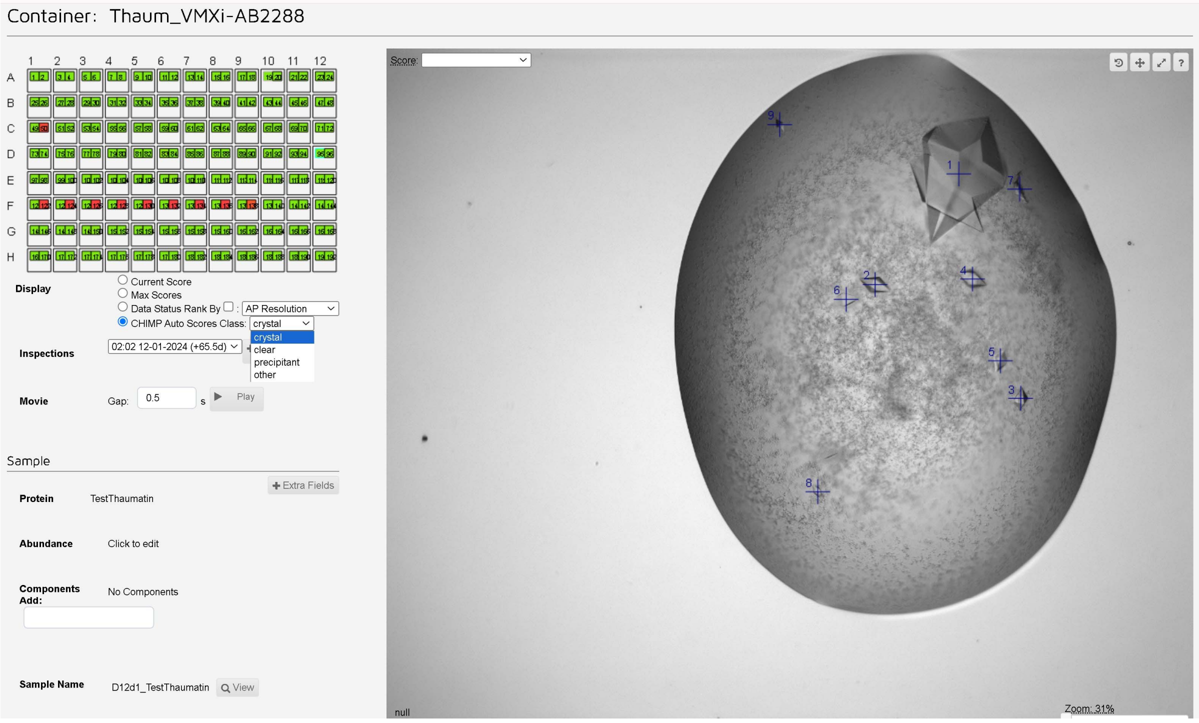

Figure S3 Image of the SynchWeb interface showing a schematic overview of classification outputs from the CHiMP Classifier-v2 CNN alongside an image overlaid with the crystal centroids detected by the VMXi CHiMP Detector network. In the schematic, the 96 plate wells are represented as larger boxes with the two subwells containing crystallisation droplets shown as smaller boxes within. Each subwell is assigned a colour on a gradient from red to green that represents the probability of the associated image being assigned to the class Crystals with green representing a high probability. The blue crosses on the associated image mark the crystal centroid positions that can be queued for automated data collection.

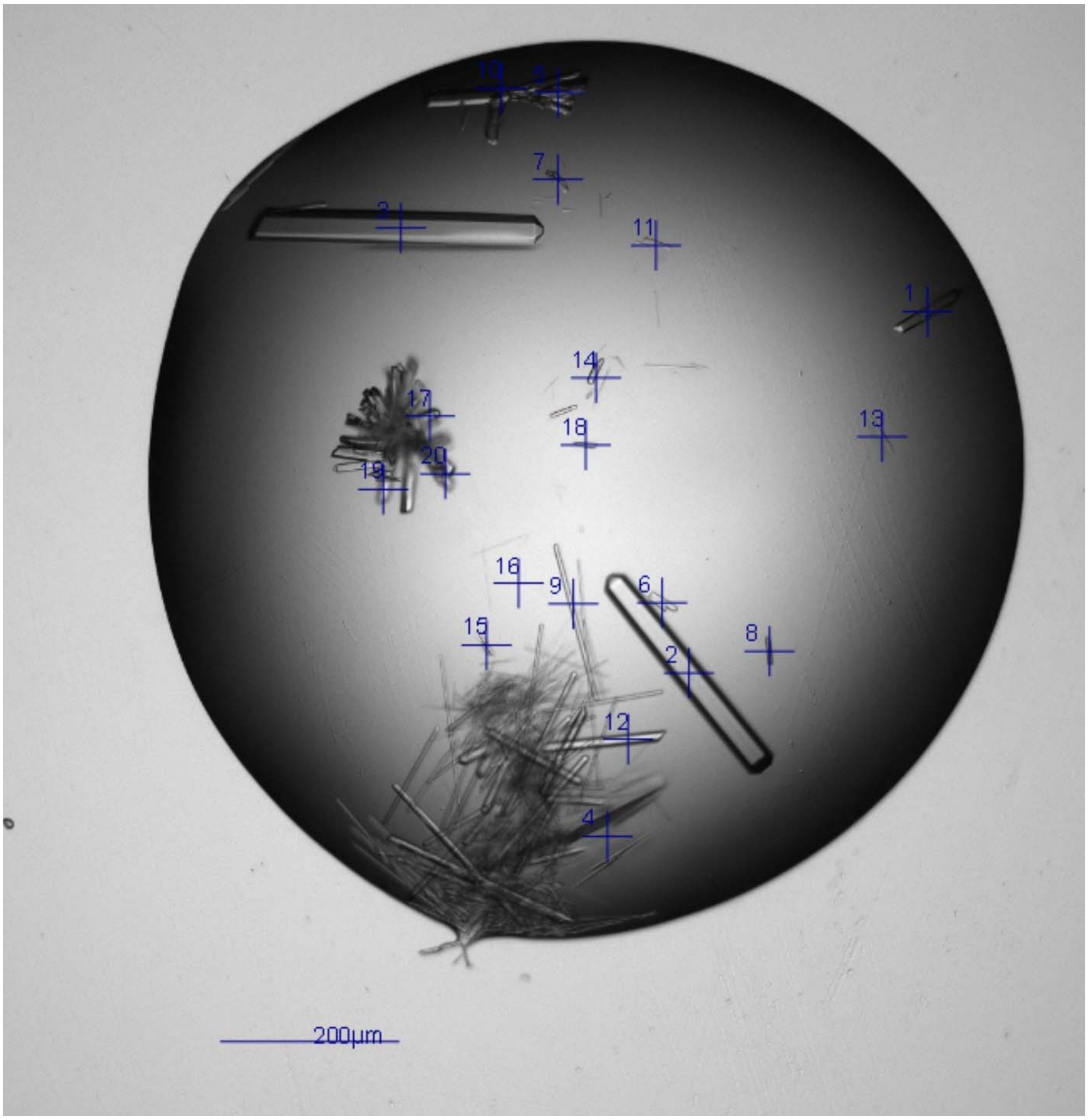

Figure S4 Image from the SynchWeb interface showing an image overlaid with the crystal centroids (blue crosses) detected by the VMXi CHiMP Detector network. The centroid coordinates are calculated from the crystal instance segmentation masks output by the network and are stored in the ISPyB LIMS.
